# Intrageneric structural variation in organelle genomes from the genus *Dystaenia* (Apiaceae): genome rearrangement and mitochondrion-to-plastid DNA transfer

**DOI:** 10.3389/fpls.2023.1283292

**Published:** 2023-12-05

**Authors:** Seongjun Park, SeonJoo Park

**Affiliations:** ^1^ Institute of Natural Science, Yeungnam University, Gyeongsan, Republic of Korea; ^2^ Department of Life Sciences, Yeungnam University, Gyeongsan, Republic of Korea

**Keywords:** plastid genome, mitochondrial genome, gene duplication, intracellular transfer, inversion

## Abstract

**Introduction:**

During plant evolution, intracellular DNA transfer (IDT) occurs not only from organelles to the nucleus but also between organelles. To further comprehend these events, both organelle genomes and transcriptomes are needed.

**Methods:**

In this study, we constructed organelle genomes and transcriptomes for two *Dystaenia* species and described their dynamic IDTs between their nuclear and mitochondrial genomes, or plastid and mitochondrial genomes (plastome and mitogenome).

**Results and Discussion:**

We identified the putative functional transfers of the mitochondrial genes *5′ rpl2, rps10, rps14, rps19,* and *sdh3* to the nucleus in both *Dystaenia* species and detected two transcripts for the *rpl2* and *sdh3* genes. Additional transcriptomes from the Apicaceae species also provided evidence for the transfers and duplications of these mitochondrial genes, showing lineage-specific patterns. Intrageneric variations of the IDT were found between the *Dystaenia* organelle genomes. Recurrent plastid-to-mitochondrion DNA transfer events were only identified in the *D. takeshimana* mitogenome, and a pair of mitochondrial DNAs of plastid origin (MIPTs) may generate minor alternative isoforms. We only found a mitochondrion-to-plastid DNA transfer event in the *D. ibukiensis plastome*. This event may be linked to inverted repeat boundary shifts in its plastome. We inferred that the insertion region involved an MIPT that had already acquired a plastid sequence in its mitogenome via IDT. We propose that the MIPT acts as a homologous region pairing between the donor and recipient sequences. Our results provide insight into the evolution of organelle genomes across the family Apiaceae.

## 1 Introduction

*Dystaenia* Kitag. is a genus of perennial herbs in the family Apiaceae (also known as Umbelliferae), which is endemic to Japan and Korea. It has two species: *D. ibukiensis* (Y.Yabe) Kitag. and *D. takeshimana* (Nakai) Kitag. (Kitagawa, 1937). This genus has attracted the attention of biologists because of its evolutionary patterns and processes in oceanic islands (Pfosser et al., 2005). This is because *D. takeshimana* is native to Ulleungdo, an oceanic island in Korea, and likely originated via anagenetic speciation from the Japanese species *D. ibukiensis* (Pfosser et al., 2005). Oceanic islands exhibit two modes of speciation: anagenesis and cladogenesis (Takayama et al., 2015). Among the oceanic islands, Ulleungdo has a higher level of endemism derived via anagenetic (88%) than cladogenetic speciation (Stuessy et al., 2006). Molecular phylogenetic studies based on nuclear internal transcribed spacer (ITS) and *trnL-F* regions have shown that *Dystaenia* is a monophyletic group, but its sister group remains unknown (Choi et al., 1998; Pfosser et al., 2005). Multilocus datasets from three genomic sequences are required to better understand the evolutionary history of *Dystaenia*.

Recently, next-generation sequencing platforms have generated deep coverage enabling the assembly of plastid and mitochondrial genomes. Comparative genomics of organelles helps shed new light on evolutionary events across the tree of life and provides valuable sources for phylogenetic studies. The plastid genome (plastome) of angiosperms generally has a conserved quadripartite structure with a pair of inverted repeat (IR), large single-copy (LSC), and small single-copy (SSC) regions (Ruhlman and Jansen, 2021). Angiosperm plastomes range from 120 kb to 170 kb in length and contain 79 proteins, 30 transfer RNA (tRNA), and four ribosomal RNA (rRNA) genes. In contrast, the mitochondrial genome (mitogenome) of angiosperms exhibits a dynamic structure with circular, linear, and branched molecules (Sloan, 2013). Angiosperm mitogenomes range from 222 kb to 983 kb in length and contain 41 proteins, 14 tRNA, and three rRNA genes (Mower et al., 2012). Furthermore, organellar phylogenomic analysis based on genome-scale data provides new insights into the origins of species that have undergone complex evolutionary histories with incomplete lineage sorting and hybridization (Liu et al., 2019; Park and Park, 2020; Wu et al., 2022).

The family Apiaceae comprises approximately 3,575–3,820 species in 442–466 genera with a cosmopolitan distribution (Christenhusz and Byng, 2016; Plunkett et al., 2018). This family includes many economically important medicinal species and exhibits extensive morphological diversity (Kljuykov et al., 2019; Wang et al., 2022). The family Apiaceae is classified into four subfamilies: Apioideae, Azorelloideae, Mackinlayoideae, and Saniculoideae. The Apioideae is the largest subfamily of Apiaceae, which contains approximately 84% of species and 85% of genera (Wen et al., 2021). Phylogenetic analyses based on nuclear ribosomal and plastid DNA sequences have identified 21 tribes and 20 clades (Downie et al., 2010). Plastid phylogenomic analysis has revealed a well-resolved relationship within the Apioideae subfamily (Wen et al., 2021). However, previous studies have shown incongruence between topologies based on chloroplast and nuclear sequence datasets (Zhou et al., 2009; Downie et al., 2010). Chloroplast capture resulting from hybridization or introgression has been suggested as a possible reason for phylogenetic incongruence (Rieseberg and Soltis, 1991; Stegemann et al., 2012). Whole-genome duplication and hybridization are involved in the evolutionary history of this family (Ren et al., 2018; Jia et al., 2023). Numerous nuclear single-copy genes from transcriptome data have been utilized to conduct a phylogenetic analysis of Apioideae, showing that this family has undergone a rapid evolutionary divergence and incomplete lineage sorting (Wen et al., 2020). To date, the complete plastomes of 439 species from 83 genera have been sequenced [National Center for Biotechnology Information (NCBI), accessed 26 June 2023], 421 species from the subfamily Apioideae, 16 species from the subfamily Saniculoideae, one species from the subfamily Azorelloideae, and one species from the subfamily Mackinlayoideae. The published plastomes of Apiaceae range from 141 kb to 179 kb and contain a full set of protein-coding genes. The plastomes have a conserved quadripartite structure, but the IR boundaries have shifted during Apiaceae genome evolution (Wang et al., 2021a; Wen et al., 2021; Yuan et al., 2021; Samigullin et al., 2022). The most notable feature among Apiaceae plastomes is the plastid DNA of mitochondrial origin (PLMT). Mitochondrion-to-plastid DNA transfers have been reported in five lineages of this family: *Crithmum* and *Petroselinum* (Downie and Jansen, 2014; Downie and Jansen, 2015) and *Caucalis*, *Cuminum*, and *Daucus* (Iorizzo et al., 2012; Spooner et al., 2017). In contrast to plastomes, there are thus far only five species from the subfamily Apioideae with sequenced mitogenomes: *Apium graveolens* (Cheng et al., 2021), *Bupleurum chinense* (Qiao et al., 2022), *Coriandrum sativum* (Wang et al., 2021b), *Daucus carota* (Iorizzo et al., 2012), and *Foeniculum vulgare* (Palumbo et al., 2020). These mitogenomes range from 281 kb to 435 kb in size, with circular molecules. Losses of six mitochondria-encoded genes (*rpl2*, *rps10*, *rps14*, *rps19*, *sdh3*, and *sdh4*) were observed in all five mitogenomes.

As part of our ongoing research on the evolution of the genus *Dystaenia*, we determined the plastid and mitochondrial genomes of two species: *D. ibukiensis* and *D. takeshimana*. A comparison of the two-organelle genomes revealed intrageneric variations in size, structure, and gene content in *Dystaenia*. We demonstrated the functional replacement of *rpl2*, *rps10*, *rps14*, *rps19*, and *sdh3* from the mitochondria to the nucleus and also compared intracellular DNA transfer (IDT) between the plastid and mitochondrial genomes. Interestingly, we identified a mitochondrion-to-plastid DNA transfer event in the *D. ibukiensis* plastome and discussed the evolutionary history of PLMT. Our results provide new insights into the evolution of Apiaceae organelle genomes, including intercompartmental transfers.

## 2 Materials and methods

### 2.1 DNA/RNA extraction and sequencing

Fresh leaves of *D. takeshimana* and *D. ibukiensis* were collected from Ulleungdo, Geongbuk, South Korea, and Mt. Ibuki, Shiga, Japan, respectively. Total genomic DNAs were isolated using the Exgene Plant SV Mini Kit (GenAll, Seoul, South Korea), following the manufacturer’s protocol. Total RNA was isolated, as described by Breitler et al. (2016). After gel electrophoresis and qualitative assessment, DNA and RNA samples were sequenced using the Illumina HiSeq2500 platform (Illumina, San Diego, CA, USA). Approximately 43 Gb and 20 Gb of 150-bp paired-end (PE) reads were generated from 550-bp insert libraries from the DNAs of *D. takeshimana* and *D. ibukiensis*, respectively. The DNAs from *D. takeshimana* and *D. ibukiensis* were used for long reads generated from four and two flow cells on the Oxford Nanopore Technologies (ONT) GridION platform (ONT, Oxford, UK), and 68 Gb and 12 Gb of ONT reads were produced, respectively. RNAs from *D. takeshimana* and *D. ibukiensis* were sequenced by the Illumina platform, generating 12 Gb and 8 Gb of 150-bp PE reads, respectively.

### 2.2 Organelle genome assembly, finishing, and annotation

Multiple assemblies for both species were generated using Canu v2.2 (Koren et al., 2017), MaSuRCA v4.0.5 (Zimin et al., 2017), SPAdes v3.15.3 (Antipov et al., 2016), and Velvet v1.2.10 (Zerbino and Birney, 2008) based on long- and short-read data. For example, single-type platform assemblies have been created using Canu for ONT reads and Velvet for Illumina reads. The default parameters were used for Canu. We used pairwise combinations of *k*-mers (99–145) and expected coverage values (50, 100, 150, 200, 500, and 1,000) without the scaffolding option for Velvet assemblies. Hybrid assemblies were generated using MaSuRCA and SPAdes, combining Illumina and ONT reads. The default parameters were used for MaSuRCA. For the SPAde assemblies, independent runs were executed with multiple coverage cutoffs (10, 50, 100, 200, and 500) using the “careful” option. All *de novo* assemblies were performed on a 64-core Linux workstation with 2,048 GB of memory. Plastid and mitochondrial contigs were identified using a BLAST-like algorithm in Geneious Prime 2022.2 (www.geneious.com) with *Liriodendron tulipifera* plastome and mitogenome sequences (NC_008326 and NC_021152) as queries. The identified organellar contigs were manually aligned, and a consensus genome sequence was generated for each by tracking and end-inspecting the organellar contigs. The coverage depth of the whole plastome and mitogenome sequences was evaluated by mapping Illumina PE and ONT reads using Bowtie v2.4.2 (Langmead and Salzberg, 2012) and BWA v0.7.17 (Li, 2013). To predict all tRNA genes in the organelle genomes, ARAGORN v1.2.38 (Laslett and Canback, 2004) and tRNAscan-SE v2.0.9 (Chan et al., 2021) were used. Circular or linear organellar genomes were generated using OGDRAW v1.3.1 (Greiner et al., 2019). The newly sequenced genomes were deposited in GenBank with accession numbers OR231235-OR231238.

### 2.3 Transcriptome assembly

Rcorrector v1.0.4 (Song and Florea, 2015) was used to correct sequencing errors in raw reads from *D. takeshimana* and *D. ibukiensis* RNA sequencing (RNA-seq). Two *Dystaenia* transcriptomes were assembled *de novo* using Trinity v2.13.2 (Grabherr et al., 2011) with the “trimmomatic” option. The completeness of the assemblies was examined using Benchmarking Universal Single-Copy Orthologs (BUSCO) v5.2.2 (Manni et al., 2021) with the lineage “eudicots_odb10” (2,326 orthologs; 2019-11-20). RNA-seq data were obtained from the NCBI Sequence Read Archive for *Apium graveolens* (SRR1023730), *Bupleurum chinense* (SRR8863755), *Coriandrum sativum* (SRR8863732), and *Saposhnikovia divaricate* (SRR8863754); four additional transcriptomes were assembled as described above.

### 2.4 Comparative organellar analyses

Repetitive sequences in *Dystaenia* organelle genomes were identified using ROUSFinder.py (Wynn and Christensen, 2019). The two plastomes and two mitogenomes were aligned using the “progressive Mauve algorithm in Mauve v2.3.182 (Darling et al., 2004) in Geneious Prime. Organellar protein-coding genes were collected from five species, each with available genomes. Individual gene alignments were generated using the “Translation Align” approach with MAFFT v7.49 in Geneious Prime. A maximum likelihood (ML) tree was constructed from a concatenated alignment of the 24-gene dataset using IQ-TREE2 v2.2.03, with a best-fit model (-m TEST) and 1,000 ultra-fast bootstrap replicates (-B 1000).

### 2.5 Identification of intracellular DNA transfer

To investigate PLMTs and mitochondrial DNAs of plastid origin (MIPTs), we performed a reciprocal “BLASTN” searches between the plastid genome and its mitochondrial counterpart with an *e*-value cutoff of 1 × 10^−6^, requiring at least 80% sequence identity and a minimum length of 50 bp. In addition, the CENSOR web server (Kohany et al., 2006) was used to search the mitochondrial genomes for putative nuclear transposable elements (TEs) with default parameters and “green plants” as a reference sequence source. To identify functional intracellular gene transfer (IGT) to the nucleus, candidate transcripts were verified by BLASTN searches for organellar genes against their transcriptomes. The conserved domain of the predicted open reading frame (ORF) was annotated by CD searches against the Conserved Domain Database (CDD) v3.19 (Lu et al., 2019). LOCALIZER v1.0.4 (Sperschneider et al., 2017) and TargetP v2.0 (Almagro Armenteros et al., 2019) were used to predict the presence of N-terminal presequences [chloroplast transit peptide (cTP) and mitochondrial targeting peptide (mTP)] and their potential cleavage sites. The transcript sequences were used as queries in “BLASTN” against the *de novo* genome sequence of two *Dystaenia* species. The sequenced genes were deposited in GenBank (**Supplementary Table 1**). Phylogenetic trees were constructed using ML methods as described above.

## 3 Results

### 3.1 Plastomic structure and gene content of *Dystaenia*

**Figure 1:**
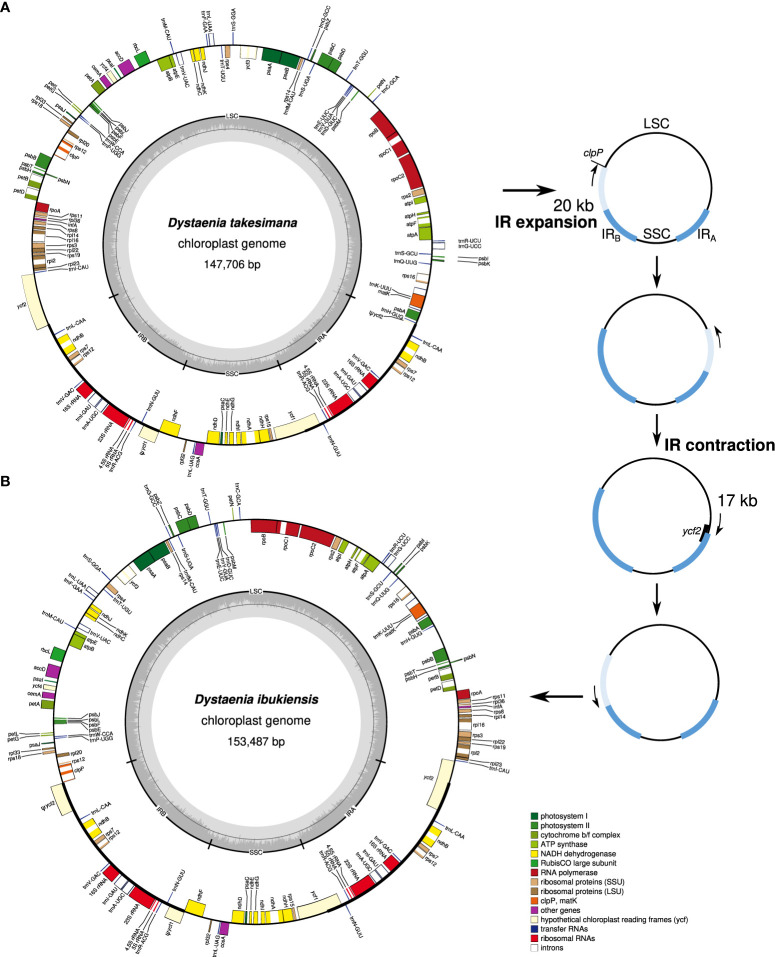
Plastid genome maps of *Dystaenia*. **(A)** *D. takeshimana.* **(B)** *D. ibukiensis*. Genes on the inside and outside of the map are transcribed in clockwise and counterclockwise directions, respectively. The hypothetical model of plastome rearrangement by IR expansion and contraction in the genus *Dystaenia* (right).

The plastomes of *D. takeshimana* (147,706 bp) and *D. ibukiensis* (153,487 bp) were assembled into a typical quadripartite structure, with the LSC and SSC separated by two IRs (**Figure 1**). Of the two species, *D. takeshimana* had the largest LSC (93,013 bp) and the smallest IR (18,568 bp). The average coverage of *D. takeshimana* and *D. ibukiensis* plastomes was 3,994× and 771× for Illumina and 5,450× and 420× for ONT, respectively (**Table 1**, **Supplementary Figure 1**). The *D. takeshimana* plastome showed syntenic conservation with the other angiosperms, whereas the *D. ibukiensis* plastome showed inversion and relocation (**Figure 1**). Mauve alignment of the two *Dysteania* species revealed three locally collinear blocks with two breakpoints (**Supplementary Figure 2A**). Both plastomes contained the same set of genes encoding 79 proteins, 30 tRNAs, and four rRNAs (**Figure 1**, **Table 1**). The average guanine-cytosine (GC) content in the *D. takeshimana* and *D. ibukiensis* plastomes was 37.5% and 37.7%, respectively, and contained six and seven small repeat pairs (<100 bp), covering 0.1% and 0.3% of the *D. takeshimana* and *D. ibukiensis* plastomes, respectively (**Supplementary Table 2**).

**Table 1:** Characteristics of *Dystaenia* organelle genomes.

	Plastome		Mitogenome	
	*D. takeshimana*	*D. ibukiensis*	*D. takeshimana*	*D. ibukiensis*
Genome size (bp)	147,706	153,487	282,211	281,432
LSC (bp)	93,013	91,719	-	–
IR (bp)	18,568	22,090	-	–
SSC (bp)	17,557	17,588	–	–
GC content (%)	37.5	37.7	–	–
Protein genes	79	79	35	35
rRNA genes	4	4	3	3
tRNA genes	30	30	13/5	13/5
Introns				
*cis*-spliced	20	20	19	19
*trans*-spliced	1	1	5	5
Repeats (bp)	208	404	94,545	71,171
PLMTs (bp)	0	2,411	–	–
MIPTs (bp)	–	–	29,289	15,731
Coverage (Illumina/ONT)	3,994/5,450	771/420	335/475	313/186

LSC, large single copy; IR, inverted repeat; SSC, small single copy; rRNA, ribosomal RNA; tRNA, transfer RNA; PLMTs, plastid DNAs of mitochondrial origin; MIPTs, mitochondrial DNAs of plastid origin; ONT, Oxford Nanopore Technologies.

### 3.2 Mitogenomic structure and gene content of *Dystaenia*

**Figure 2:**
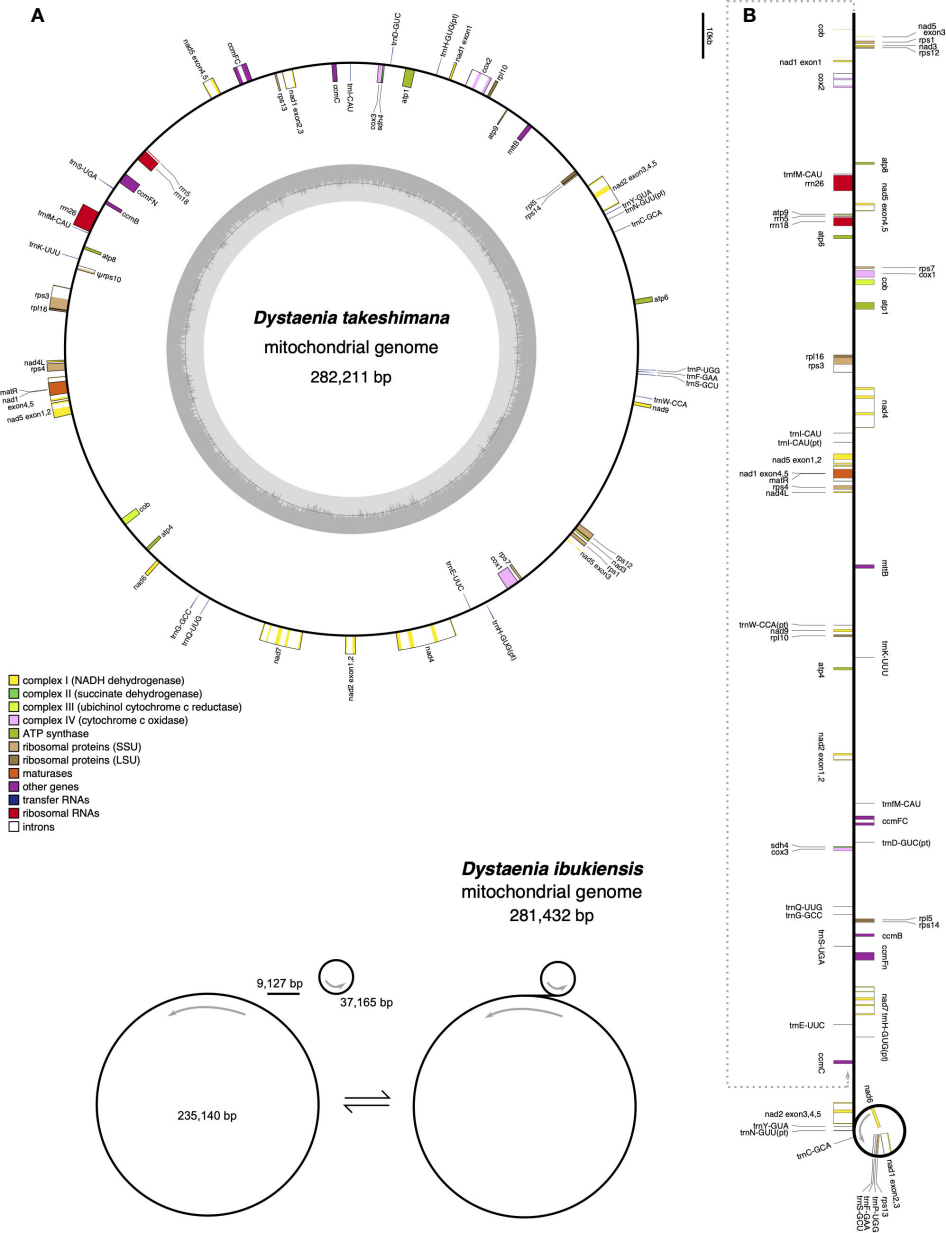
Mitochondrial genome maps of *Dystaenia*. **(A)** *D. takeshimana.* **(B)** *D. ibukiensis.* Genes on the inside and outside of the map are transcribed in clockwise and counterclockwise directions, respectively.

**Figure 3:**
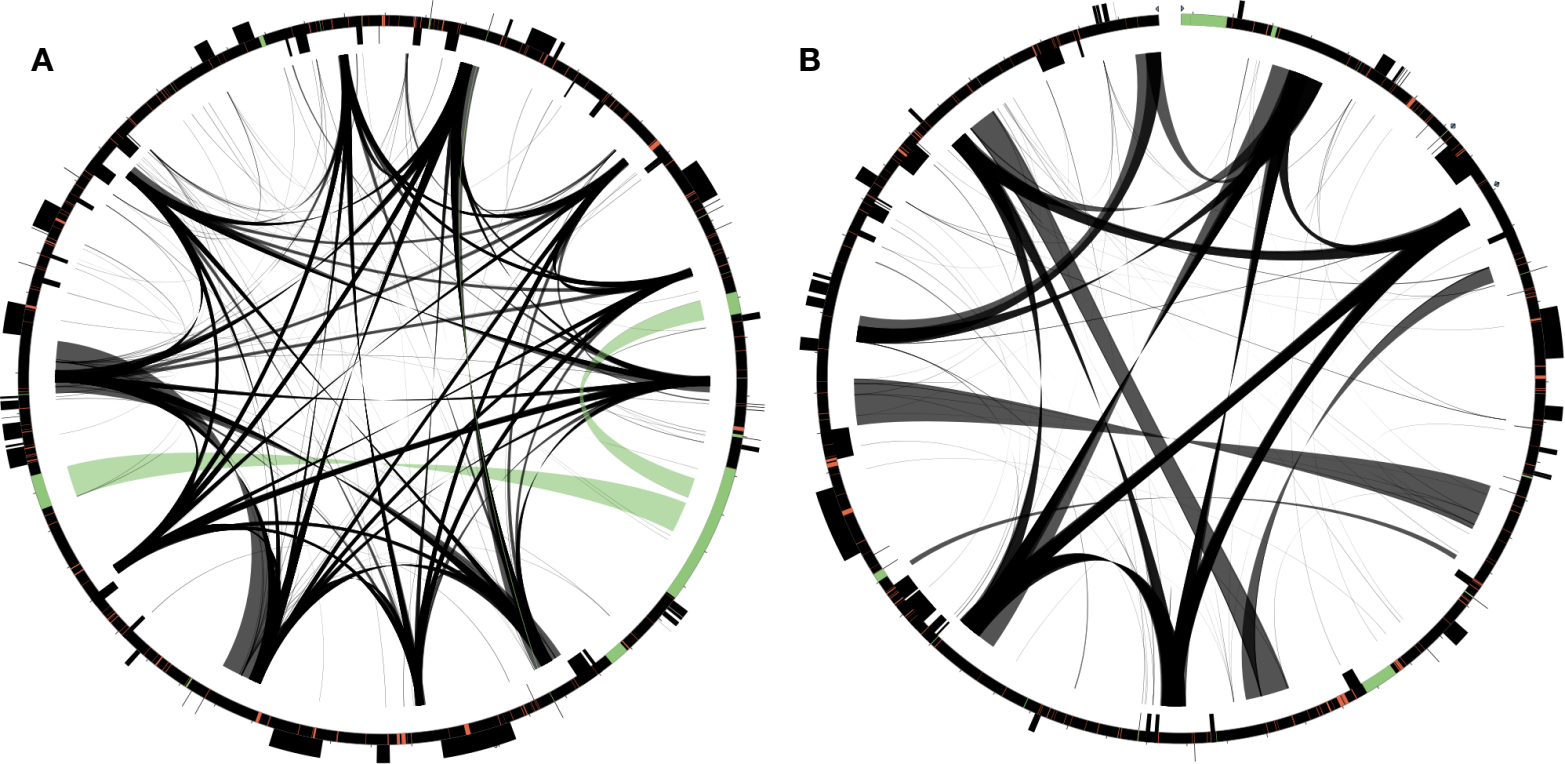
Distribution of repetitive DNA in two *Dystaenia* mitogenomes. **(A)** *D. takeshimana*. **(B)** *D. ibukiensis*. Black lines within circular maps indicate the positions of the pairs of repeats, with crossed connecting lines denoting reverse repeats. Black boxes on the inner and outer circle indicate the positions of mitochondrial genes. Green and red lines inside of the circular maps indicate mitochondrial DNAs of plastid origin and transposable elements, respectively.

The *D. takeshimana* mitogenome (282,211 bp) was assembled into a single circular molecule (**Figure 2A**). In contrast, the *D. ibukiensis* mitogenome (281,432 bp) was assembled into two circular molecules (235,140 bp and 37,165 bp) with a linear molecule (9,127 bp) connected to the two circles (**Figure 2B**). The average coverage of the *D. takeshimana* and *D. ibukiensis* mitogenomes was 335× and 313× for Illumina and 475× and 186× for ONT, respectively (**Table 1**, **Supplementary Figure 1**). Comparative analysis revealed significant structural variation between the two mitogenomes (**Supplementary Figure 2B**), although 91% of the *D. takeshimana* mitogenome was homologous to the *D. ibukiensis* mitogenome. However, two mitogenomes shared a loss of synteny, in which eight of the 14 ancestral gene clusters were missing (**Supplementary Figure 2C**). Both mitogenomes contained the same set of genes encoding 35 proteins, 18 tRNAs (including five plastid-derived tRNAs), and three rRNAs (**Figure 2**, **Table 1**). Both species lack four genes, 5′ ribosomal protein subunits L2 (*rpl2*), S10 (*rps10*), S19 (*rps19*), and succinate dehydrogenase 3 (*sdh3*), in their mitogenomes. In addition, both mitogenomes had a truncated *sdh4* gene at the C-terminus, with only a portion of the conserved domain. The ribosomal protein subunit L5 (*rpl5*) gene was truncated at the N-terminus in only *D. ibukiensis* mitogenome. The average GC contents from *D. takeshimana* and *D. ibukiensis* mitogenomes were 44.6% and 44.3%, respectively. Two mitogenomes contained 79 and 55 repeat pairs, covering 33.5% and 25.3% of the *D. takeshimana* and *D. ibukiensis* mitogenomes, respectively (**Figure 3**, **Supplementary Table 3**). The *D. takeshimana* mitogenome contained 20 large (> 1,000 bp) repeats, ranging from 1,034 bp to 5,706 bp, and the *D. ibukiensis* mitogenome contained 13 large repeats, ranging from 2,140 bp to 6,502 bp (**Supplementary Table 3**).

### 3.3 Migration of organelle genes into the nuclear genomes of *Dystaenia*

Although losses of the mitochondrial-encoded 5′ *rpl2*, *rps10*, *rps19*, and *sdh3* genes were only identified in the *Dystaenia* organelle genomes, we performed BLASTN searches using 41 mitochondrial and 79 plastid gene sequences against each transcriptome dataset. BUSCO assessment of the transcriptomes resulted in 83.6% (*D. takeshimana*) and 62.1% (*D. ibukiensis*) (**Supplementary Figure 3**). We identified multiple transcripts with high sequence identities to the four genes containing the targeting sequences and conserved domain (**Table 2**, **Supplementary Figure 4**). Moreover, we identified a nuclear transcript for mitochondrial *rps14*, which is present in the mitogenome (**Table 2**, **Supplementary Figure 4**). In particular, two nuclear transcripts for the mitochondrial 5′ *rpl2* and *sdh3* were identified in both *Dystaenia* transcriptomes (**Table 2**, **Supplementary Figure 4**). To test the potential gene split transfer of truncated *sdh4*, we performed BLASTN searches using *Amborella* mitochondrial *sdh4* as the query. One and two transcripts were identified in the *D. takeshimana* and *D. ibukiensis* transcriptome, respectively (**Supplementary Figure 4**). These transcripts contained a portion of the conserved peptidylprolyl isomerase (PPIase) domain upstream of the *sdh4* domain. However, TargetP failed to predict the mitochondrial target sequences. No nuclear transcript was identified for the truncated mitochondrial *rpl5* gene from the *D. ibukienesis* mitogenome.

**Table 2:** Transit peptide prediction of nuclear-encoded ORFs.

Gene	Species		TargetP-2.0				Predotar v1.04		LOCALIZER 1.0.4			
		length (aa)	cTP	mTP	Probability	Tplen	Plastid	Mitochondrial	mTP		cTP	
*5’ RPL2*	*D. takeshimana1*	294	0.0005	**0.9431**	0.5908	37	0	**0.84**	**0.998**	21	–	–
	*D. takeshimana2*	294	0.0027	**0.8953**	0.6081	37	0.02	**0.82**	**0.996**	21	–	–
	*D. ibukiensis1*	293	0.0135	**0.9220**	0.5901	37	0.06	**0.77**	**0.996**	21	–	–
	*D. ibukiensis2*	293	0.0030	**0.8895**	0.5453	37	0.02	**0.74**	**0.997**	24	–	–
*RPS10*	*D. takeshimana*	235	0.2708	**0.6420**	0.1745	41	0.01	**0.85**	**0.974**	21	–	–
	*D. ibukiensis*	235	0.2557	**0.6422**	0.1561	92	0	**0.9**	**0.967**	42	–	–
*RPS14*	*D. takeshimana1*	142	0.1369	**0.7531**	0.4505	40	0.02	**0.8**	**0.993**	34	**0.89**	24
	*D. takeshimana2*	133	0.0469	0.3619	–	–	0.09	0.02	–	–	–	–
	*D. ibukiensis*	142	0.1357	**0.7411**	0.314	40	0.02	**0.8**	**0.993**	34	**0.89**	24
*RPS19*	*D. takeshimana*	149	0.1038	**0.6978**	0.3311	32	0.03	**0.27**	–	–	**0.994**	32
	*D. ibukiensis*	149	0.1016	**0.7013**	0.3293	32	0.01	**0.27**	–	–	**0.994**	32
*SDH3*	*D. takeshimana1*	251	0.0005	**0.9966**	0.3903	28	0	**0.8**	**0.996**	28	–	–
	*D. takeshimana2*	276	0.0000	**0.9999**	0.86	24	0	**0.84**	**0.999**	25	–	–
	*D. ibukiensis1*	251	0.0003	**0.9969**	0.4073	28	0	**0.8**	**0.995**	28	–	–
	*D. ibukiensis2*	276	0.0000	**0.9999**	0.86	24	0	**0.84**	**0.999**	25	–	–

cTP, chloroplast transit peptide; mTP, a mitochondrial targeting peptide. Tplen means predicted presequence length (cleavage sites). Bold font indicates prediction of localization (chloroplast or mitochondrion).

**Figure 4:**
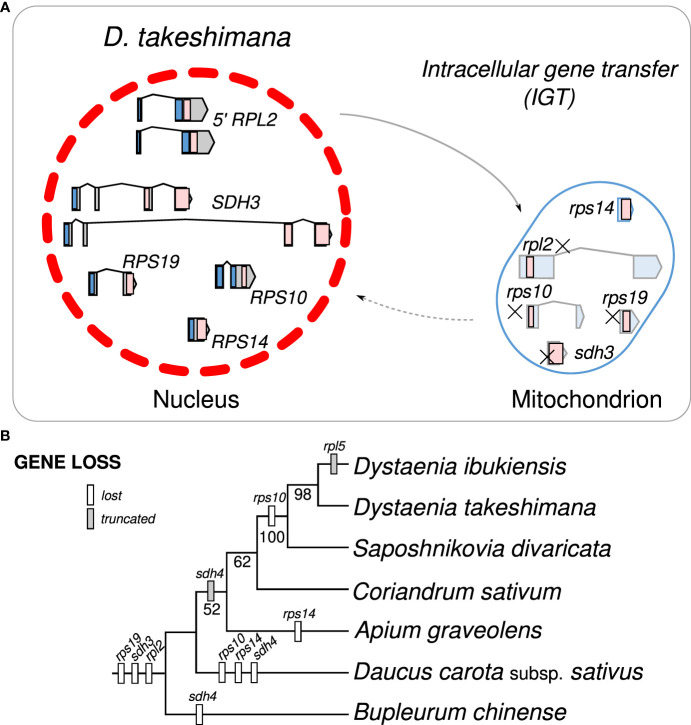
Intracellular gene transfer events to the nucleus in *Dystaenia* with related species. **(A)** Schematic diagram of mitochondrial gene transfers from mitochondrial genome to the *D. takeshimana* nuclear genome. **(B)** Phylogenetic distribution of gene content among seven Apiaceae species.

**Figure 5:**
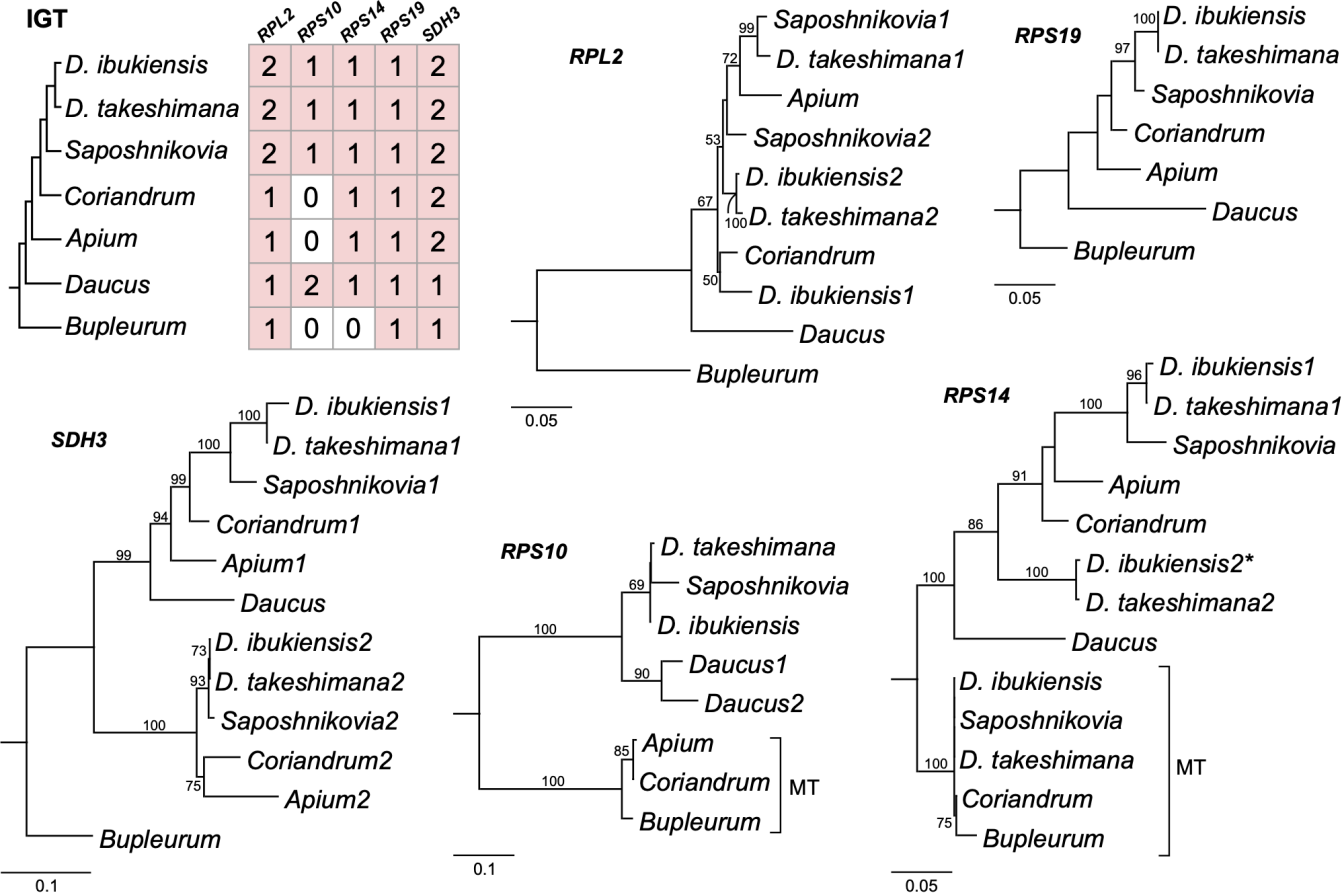
Phylogenetic trees for the nuclear-encoded genes from seven Apiaceae species. A species phylogeny and copy number variation in each nuclear-encoded genes (right). Maximum-likelihood trees based on each nuclear gene. The numbers “1” and “2” after each species in the phylograms represent paralogs. Bootstrap support values > 50% are shown at nodes. MT, mitochondrial gene; asterisk, pseudogene.

Examining the draft *D. takeshimana de novo* genome assembly identified the generic structure of the nuclear genes (**Figure 4A**). This analysis revealed several exons and introns in the five nuclear genes. The nuclear-encoded *RPS14* is intronless, whereas the 5′ *RPL2*, *RPS10*, and *RPS19* contain two exons, whereas *SDH3* contains four exons (**Figure 4A**). Variations in the intron length of each 5′ *RPL2* and *SDH3* gene were identified (**Figure 4A**). In the case of the nuclear-encoded *RPS14*, we found extra copies with 70.9% and 65.3% nucleotide sequence identity on the other *D. takeshimana* and *D. ibukiensis* nuclear genome scaffolds, respectively. The *D. takeshimana* nuclear genome contains an intact ORF; the *D. ibukiensis* nuclear genome has multiple internal stop codons. We constructed a phylogenetic tree using 24 mitochondrial genes from seven available Apiaceae mitogenomes (**Figure 4B**). The phylogenetic distribution of gene loss showed that all analyzed species shared the loss of mitochondrial *rpl2*, *rps19*, and *sdh3* genes. The loss of *sdh4* occurred independently in *D. carota* subsp. *sativus* and *B. chinense*, and loss of *rps10* occurred independently in *D. carota* subsp. *sativus* and in the two *Dystaenia* species/*S. divaricate* clade. The loss of *rps14* occurred independently in *A. graveolens* and *D. carota* subsp. *sativus*. The truncation of *sdh4* occurred in the common ancestor of *A. graveolens*, *C. sativum*, the two *Dystaenia* species, and *S. divaricate*. The truncation of *rpl5* is unique to the *D. ibukiensis* mitogenome. Comparison of the five high-quality transcriptomes of *A. graveolens*, *B. chinense*, *C. sativum*, *D*. *carota* subsp. *sativus*, and *S. divaricate* (**Supplementary Figure 3**) provided additional evidence for multiple gene transfers from the mitochondria to the nucleus (**Figure 5**, **Supplementary Table 4**). Similar to the two *Dystaenia* species, *S. divaricate* contains two nuclear transcripts for 5′ *RPL2*, and *A. graveolens*, *C. sativum*, and *S. divaricate* contain two copies of the *SDH3* transcripts (**Figure 5**). Two copies of *RPS10* are unique to *D*. *carota* subsp. *sativus*. One transcript for 5′ *RPL2*, *RPS14*, and *RPS19* was found in the *A. graveolens*, *C. sativum*, and *D*. *carota* subsp. *sativus* transcriptomes. The *B. chinense* transcriptome contains *5*′ *RPL2*, *RPS19*, and *SDH3.* Similar to the two *Dystaenia* species, multiple transcripts of mitochondrial *sdh4*, which also contains a portion of the conserved peptidylprolyl isomerase (PPIase) and *sdh4* domains without mitochondrial targeting sequences, were found in three transcriptomes (*A. graveolens*, *B. chinense*, and *D*. *carota* subsp. *Sativus*; **Supplementary Figure 5**). However, transcripts from *C. sativum* and *S. divaricate* transcriptomes included a 5′ extension of 113 bp and 126 bp, respectively, with an incomplete ORF at the N-terminus. The first 32 and 42 amino acids of these ORFs (when “ATA” is translated as a start codon) were predicted as an mTP by LOCALIZER (0.974 and 0.889).

We further generated a phylogenetic tree of each gene (5′ *RPL2*, *RPS10*, *RPS14*, *RPS19*, and *SDH3*) (**Figure 5**). The phylograms based on the 5′ *RPL2* and *RPS10* matrix conflicted with the 24-gene data matrix of the phylogenetic tree. However, the phylogenetic analyses of *RPS19*, *SDH3*, and *RPS14* were consistent with the 24-gene data matrix of the phylogenetic tree, although *A. graveolens* and *C. sativum* were conflicting. Although it was difficult to infer the evolutionary history of the gene duplication events for the 5′ *RPL2*, it was possible for the *SDH3* and *RPS14* based on robust relationships with high bootstrap values. For example, phylogenetic analysis of the nuclear-encoded *SDH3* copies suggested that duplication events occurred in the common ancestors of *Dystaenia*, *A. graveolens*, *C. sativum*, *D*. *carota* subsp. *sativus*, and *S. divaricate*, and the loss of one copy occurred independently in *D*. *carota* subsp. *sativus* (**Figures 4**, **5**). Phylogenetic analysis of the nuclear-encoded *RPS14* copies suggested that the IGT occurred in the common ancestor of the two *Dystaenia* species, *A. graveolens*, *C. sativum*, *D*. *carota* subsp. *sativus*, and *S. divaricate*, and then stochastic losses occurred after the duplication events (**Figures 4**, **5**).

### 3.4 Migration of plastid and nuclear DNA into the mitogenome of *Dystaenia*

The *D. takeshimana* and *D. ibukiensis* mitogenomes contained 29,079 bp and 12,684 bp of MIPTs (**Table 1**, **Supplementary Table 5**), accounting for 10.3% and 4.5% of each mitogenome, respectively. MIPTs were widely scattered across mitogenomes (**Figure 3**). The 16 insertion regions of *D. takeshimana* contained three intact protein-coding genes (*ndhB*, *rps7*, and *rps12*), three rRNAs (16S), 10 tRNAs genes (three of which had two copies), several partial genes (*ndhB*, *petG*, *psaB*, *rpoC1*, and *ycf2*), and intergenic spacer regions (**Supplementary Table 5**). The 14 insertion regions in *D. ibukiensis* contained one intact protein-coding gene (*matK*), three rRNAs (23S, 4.5S, and 5S), seven tRNAs (two of which had two copies), one pseudogene, several partial genes (*clpP*, *psbA*, *psbB*, and *rpoB*), and intergenic spacer regions (**Supplementary Table 5**). Both mitogenomes shared multiple fragments of tRNA genes (*trnD-GUC*, *trnH-GUG*, *trnI-CAU*, *trnN-GUU*, and *trnW-CCA*), partial *rpoB* and *rrn23* genes, and a large fragment (part of the *trnI-GAU*, intact *trnA-UGC*, and *rrn23* genes). The *rpoB* fragment (215 bp) from the *D. ibukiensis* mitogenome was located upstream of the truncated *rpl5* gene. In the *D. takeshimana* mitogenome, five MIPTs were associated with three repeat pairs (79 bp, 2736 bp, and 4322 bp). The small repeat pairs included *trnH-GUG*, and two large repeat pairs contained two partial fragments (a partial fragment of *ndhB*, intact *rps7*, and *rps12*; a partial fragment of *ndhB* and *ycf2* and intact *trnL-CAA*) from the largest MIPT (**Figure 3A**). In addition to plastid-derived sequences, the *D. takeshimana* and *D. ibukiensis* mitogenomes contained 16,272 bp of (5.8%) and 15,522 bp (5.5%) of TEs, respectively (**Supplementary Table 6**), the majority of which were LTR retrotransposons (50% and 52%). TEs in both mitogenomes were inserted into the genic and intergenic regions (**Figure 3**).

### 3.5 Migration of mitochondrial DNA into the plastome of *Dystaenia*

**Figure 6:**
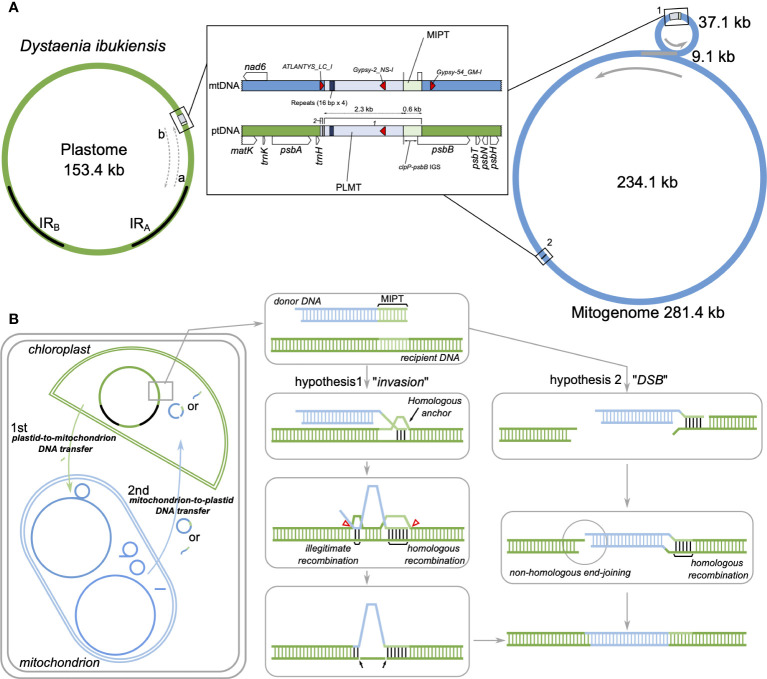
Intracellular DNA transfer events to the plastids in *Dystaenia*. **(A)** Schematic diagram of genomic regions surrounding the plastid DNA of mitochondrial origin (PLMT) and mitochondrial DNA of plastid origin (MIPT) from *D. ibukiensis* organelle genomes. Genome maps are based on the results of **Figures 1**, **2**. The IR_A_ and IR_B_ indicate inverted repeats in the plastome. **(B)** Models of intracellular DNA transfer events in *D. ibukiensis* organelle genomes. The hypothetical models illustrate the potential integration of PLMT from the mitogenome into the plastome through invasion or double-stranded break (DSB) mechanism after plastid-to-mitochondrion DNA transfer.

The regions that Mauve did not align indicated that the sequence lacked detectable homologous regions between the two plastomes (**Supplementary Figure 2A**). According to the possible scenario for IR boundary shifts, the region is associated with the end of the boundary (**Figures 1**, **6**). Nucleotide sequence alignment of the *trnH-psbB* intergenic spacer from the *D. ibukiensis* plastome with two intergenic spacers (*clpP-psbB* and *trnH-ycf1*) from the *D. takeshimana* plastome revealed the insertion of a 2,411-bp fragment. The GC content of the insertion region was 46.5%, whereas the remaining LSC regions had a GC content of 36.2%. Illumina reads from *D. ibukiensis* mapped to its plastome confirmed the insertion of *trnH* and *psbB* resulting from uniform coverage. The primers designed to amplify this region yielded PCR products of the expected size (**Supplementary Figure 6**).

To identify possible mitochondrion-to-plastid DNA transfer [plastid DNA of mitochondrial origin (PLMT)], the inserted region sequences of the *D. ibukiensis* plastome were used to query the *D. ibukiensis* mitogenome using “BLASTN” searches. BLAST (> 30 bp in length) returned two hits to the two non-coding regions of the mitogenome (**Figure 6A**, **Supplementary Figure 7**). A large fragment (2,294 bp) was located upstream from the *nad6* gene in a small mitochondrial circle with 99.3% nucleotide identity. Compared with our MIPT analysis, a 579-bp sequence adjacent to the large fragment revealed MIPT. A small fragment (49 bp) was located upstream from the *atp4* gene in a large mitochondrial circle with 100% nucleotide identity. The short sequences (10 bp, “AGAAAGGCCC”) at the end of the small fragment were identical to the sequences downstream of the *trnH* gene in the *D. takeshimana* plastome, indicating that the short sequences were *D. ibukiensis* plastid DNA. Three hits to TEs (*ATLANTYS_LC_I*, *Gypsy-2_LC_I*, and *Gypsy-54_GM-I*) were detected in the mitogenome, whereas the plastome contained only one TE (**Figure 6A**). PLMT had three repetitive sequences of “CCTACGTATGCCTATG”; however, the mitogenome contained four copies (**Figure 6A**). No PLMT hits were identified from reciprocal BLAST searches between the *D. takeshimana* plastid and mitochondrial genomes, indicating that the mitochondrion-to-plastid DNA transfer event was unique to the *D. ibukiensis* plastome.

## 4 Discussion

Plastids and mitochondria originate from cyanobacterial and alpha-proteobacterial endosymbiont ancestors within plant cells, respectively (Lang et al., 1999; Keeling, 2010). The angiosperm organelle genomes are typically assembled into circular maps (Mower et al., 2012; Smith and Keeling, 2015; Ruhlman and Jansen, 2021). However, mitogenomes often exhibit subcircular, linear, and branched chromosomes (Bendich, 2007; Sloan, 2013; Smith and Keeling, 2015). The presence of recombination activities with repeats (particularly large repeats) contribute to variations in the mitogenome structural organization (Kühn and Gualberto, 2012). Complete plastid and mitochondrial genomic sequences are required to understand organelle genome evolution better. Here, we generated the complete plastid and mitochondrial genomes of two *Dystaenia* species and showed contrasting patterns of genomic structure within organellar genome evolution. For example, compared with other Apiaceae plastomes, the *D. takeshimana* plastome was highly conserved, whereas *D. ibukiensis* underwent relocation and inversion events of a large region (17 kb, a part of *ycf2* through *psbB* genes) (**Figure 1**). We inferred that the structural changes in the *D. ibukiensis* plastome were likely caused by a series of IR expansions and contractions (**Figure 1**): first, an expansion at the IR_B_/LSC boundary to the *clpP* occurred, followed by a contraction at the IR_A_/LSC boundary, from the *clpP* to the middle of the *ycf2* gene. In the plastomes of Apiaceae, the inversion of only a small fragment (490 bp, *trnY-trnD-trnE*) has been documented in *Peucedanum* (Liu et al., 2022) and *Angelica* (Wang et al., 2021a). It is well known that the expansion and contraction of the IR play a key role in the evolution of Apiaceae plastomes. In *D. ibukiensis*, IR expansion and contraction resulted in the relocation of a large gene block (**Figure 1**). The occurrence of large events in the *D. ibukiensis* plastome is a unique feature of the family Apiaceae. In particular, the *D. ibukiensis* plastome harbored foreign DNA transferred from its mitochondrial counterpart (**Figure 6**). Incorporating foreign DNA into the *D. ibukiensis* plastome is a valuable case study for the evolution of plastomes in Apiaceae.

The *D. takeshimana* mitogenome mapped a circular molecule similar to those of Apiaceae mitogenomes (*A. graveolens*, *B. chinense*, *C. sativum*, *D. carota* subsp. *sativus*, and *F. vulgare*), whereas the *D. ibukiensis* mitogenome mapped two molecules with substoichiometric linear (**Figure 2**). Similar to *D. ibukiensis*, a recent study demonstrated that the *Aragoa cleefii* mitogenome from Plantaginaceae exhibits a high level of complexity, characterized by the presence of two circular maps connected by a substoichiometric linear (Mower et al., 2021). Large recombinogenic repeats are commonly found in plant mitogenomes. Although the *D. takeshimana* mitogenome (20) had more large repeat pairs (>1 kb) than the *D. ibukiensis* mitogenome (13), all *D. ibukiensis* repeat pairs were >2 kb. The presence of large repeats (>1 kb) in both *Dystaenia* mitogenomes indicates the existence of multiple major or minor alternative isoforms, as confirmed by contigs generated from hybrid assemblies of short and long reads. Therefore, additional chromosomal configuration may be present in the two *Dystaenia* mitochondria. Interestingly, we observed the same fragments of plastid-derived sequences in the *D. takeshimana* mitogenome (**Figure 3**), identifying a pair of repeats. The three MIPTs can be mediators of homologous recombination in the *D. takeshimana* mitogenome. It is likely that the fragments moved independently from the plastome and were inserted into the mitogenome or that two large fragments of plastid sequences were inserted into the mitogenome, one of which was split by genomic recombination.

Our analyses showed that gene loss and transfer to the nucleus during the evolution of Apicaeae organelles occurred only in mitogenomes. In angiosperm mitogenomes, a high frequency of 15 ribosomal proteins and two *sdh* gene losses have been documented (Adams et al., 2002). In the common ancestor of eudicots, two protein-coding genes, *rps2* and *rps11*, were lost (Adams et al., 2002). The successful transfers of 10 mitochondrial genes (5′ *rpl2*, *rpl5*, *rps4*, *rps7*, *rps10*, *rps12*, *rps14*, *rps19*, *sdh3*, and *sdh4*) into the nucleus have been reported in multiple lineages (Adams et al., 2001; Park et al., 2014; Park et al., 2015; Park and Park, 2020). Comparative analyses of *Dystaenia* and related species indicated that three mitochondrial genes (*rpl2*, *rps19*, and *sdh3*) were transferred to the nucleus in the common ancestor of all analyzed Apiaceae species; one (*rps10*) was unique to the specific lineages, *Dystaenia*/*Sposhnikovia* clades and *D. carota* subsp. *sativus*; and *rps14* was transferred to the nucleus in the common ancestor of all analyzed Apiaceae species except for *Bupleurum* (**Figures 4**, **5**). The split transfer of the 3′ end of the mitochondrial *rpl2* gene to the nucleus occurred in the common ancestor of core eudicots (Adams et al., 2001), in which the 5′ portion of *rpl2* was present in the mitogenome. Subsequently, multiple transfers of 5′ *rpl2* to the nucleus were documented in three lineages: *Medicago* Soybean (Adams et al., 2001) and *Geranium* (Park et al., 2015). We also found the 5′ *RPL2* transcripts in multiple transcriptomes from Apiaceae, resulting in the complete transfer of the mitochondrial *rpl2* to the nucleus during Apiaceae evolution. In particular, the two *Dystaenia* species and *S. divaricate* contained two copies of 5′ *RPL2* genes, and the phylogenetic analysis indicated that its origin and evolutionary history remain unclear. In the case of *RPS19*, all analyzed species contained one copy, and the topology was consistent with the ML tree based on the 24 mitochondrial gene sets, although some support values at the nodes were weak.

The phylogenetic relationships of *SDH3* among the analyzed Apiaceae lineages provide a good example of how mitochondrial genes are transferred to the nucleus and duplicated (**Figure 5**). The phylogenetic analysis of nuclear-encoded *SDH3* copies suggested that a single transfer to the nucleus occurred in the common ancestor of the seven species. Duplication events occurred in the common ancestors of *Dystaenia*, *A. graveolens*, *C. sativum*, *D. carota* subsp. *sativus*, and *S. divaricate*, and, subsequently, the loss of one copy occurred independently in *D. carota* subsp. *sativus*. The nuclear-encoded *RPS14* gene was found in all transcriptomes except *Bupleurum*, suggesting that functional replacement occurred in the common ancestors of *Dystaenia*, *A. graveolens*, *C. sativum*, *D. carota* subsp. *sativus*, and *S. divaricate*, although only the loss of mitochondrial *rps14* occurred independently in *A. graveolens* and *D. carota* subsp. *sativus*. The coexistence of mitochondrial and nuclear *rps14* homologs within *Dystaenia*, *C. sativum*, and *S. divaricate* indicates that successful functional replacement of the nucleus was necessary before the original mitochondrial copy was lost. In the case of *Dystaenia rps14*, two copies were detected in the nucleus. Phylogenetic analysis suggests that two independent transfers of mitochondrial *rps14* to the nucleus occurred in the common ancestor of *Dystaenia* and that the copies acquired a targeting sequence. However, the second copy from both species may fail to function as a replacement because the targeting sequence from the second copy of *D. takeshimana* has a weak signal (**Table 2**), and the second copy of *D. ibukiensis* loses the targeting sequence and has multiple internal stop codons. Similar to *Dystaenia*, the *Rhazya stricta* genome contains two nuclear copies of *rps14* and one mitochondrial copy; one of the nuclear copies is a pseudogene (Park et al., 2014). However, phylogenetic analysis supports a single transfer of mitochondrial *rps14* to the *Rhazya* nuclear genome (Park et al., 2014).

Although multiple transcripts for mitochondrial *sdh4* contained parts of the PPIase and *sdh4* domains, our analyses did not find sufficient evidence for mitochondrial target peptides to shuttle the product back to the mitochondria. However, the *Arabidopsis thaliana* mitogenome also contains a truncated *sdh4* gene at the C-terminus, previously annotated as a pseudogene. The nuclear-encoded *SDH4* gene presence, which contains an mTP and a partial domain of *sdh4*, suggests the possibility of split gene transfer and fission of *sdh4* in the mitogenome. MIPT was located upstream of the truncated *rpl5* gene, suggesting that the transfer event disrupted the ORF of *rpl5*, resulting in a loss of functionality. However, our analysis found no evidence for IGT of the truncated mitochondrial *rpl5* in the *D. ibukiensis* mitogenome. The evolutionary fate of mitochondrial *rpl5* in *D. ibukiensis* and the fate of mitochondrial *sdh4* among the analyzed genera were unclear from the present data. Additional deep genome sequencing will be required to address this question.

In addition to IGT to the nucleus, inter-compartmental transfers between plant organelle genomes have been documented; plastid-to-mitochondrial DNA transfers are common, but transfers in the opposite direction are rare (Mower et al., 2012). Within angiosperm plastomes, mitochondrion-to-plastid DNA transfers have been documented in five families: *Anacardium* in Anacardiaceae (Rabah et al., 2017); *Caucalis*, *Crithmum*, *Cuminum*, *Daucus*, and *Petroselinum* in Apiaceae (Iorizzo et al., 2012; Downie and Jansen, 2014; Downie and Jansen, 2015; Spooner et al., 2017), the tribe Asclepiadeae of Apocynaceae (Straub et al., 2013); *Convallaria* in Asparagaceae (Raman et al., 2019); and *Paspalum* and *Pariana* in Poaceae (Ma et al., 2015; Burke et al., 2016). Most fragments of mitochondrial DNA were inserted into their plastome-IR region, except for *Crithmum*, *Petroselinum*, and tribe Asclepiadeae. The foreign DNA of *Crithmum* and *Petroselinum* was found between the IR_A_ and LSC gene *trnH-GUG*, and the tribe Asclepiadeae plastomes contained PLMT sequences between *rps2* and *rpoC2* genes in LSC. We discovered a mitochondrial insertion in the *D. ibukiensis* plastome *trnH-psbB* intergenic spacer in the LSC and inferred that the insertion sequences were associated with IR boundary shifts (expansion and contraction) (**Figure 6**). The complete *D. ibukiensis* mitogenome provided strong evidence for the occurrence of DNA transfer into the plastid counterpart, showing that the PLMT was fragmented into two in the mitogenome (**Figure 6**). However, the mitochondrial DNA was likely to transfer intact as plant mitogenomes have a high frequency of genomic rearrangements between recombinationally active repeats. Only *D. carota* and *Asclepias syriaca* had complete mitogenomes, which confirmed that the PLMTs comprise two or three pieces in their mitogenomes, also suggesting a single DNA transfer event (Iorizzo et al., 2012; Straub et al., 2013).

The integration of mitochondrial DNA into the *D. ibukiensis* plastome can be comprehensively explained by considering the hypothesis of invasion and DSB (**Figure 6**). We propose two hypotheses to explain how the mitochondrial DNA fragment was integrated into the *D. ibukiensis* plastome (**Figure 6B**). In both hypotheses, plastid-to-mitochondrial DNA transfer occurred first, followed by mitochondrion-to-plastid DNA transfer. In this case, the transfer portion of the mitochondrial DNA could be a circular or linear molecule containing MIPT, which acts as a homologous region pairing between the donor and recipient sequences. After mitochondrial DNA is imported into the plastid, it can be integrated into the plastome by two different processes: 1) a displacement loop (D-loop) associated with IR boundary shifts facilitates pairing with a homologous anchor at an MIPT site and strand invasion to initiate the recombination repair process; and 2) fusion between the donor and recipient DNA occurs within or at the end of the interacting microhomologous sequences (illegitimate recombination), followed by the deletion of a segment of the recipient DNA. Finally, the integration of mitochondrial sequences is completed by DNA replication, and a double-strand break (DSB) occurs before the interaction with the plastome. Next, integration occurs as a single-stranded DNA molecule is annealed at an MIPT site to a complementary single-stranded overhang present at a DSB site in the plastome. Finally, integration is completed by second-strand synthesis and ligation via non-homologous end-joining.

The double D-loop strategy has been widely recognized as a fundamental aspect of plastid DNA replication (Heinhorst and Cannon, 1993). One potential mechanism that could contribute to IR expansion in the plastome was also associated with a DSB, which is subsequently followed by strand invasion, expansion, and recombination within the IR region (Goulding et al., 1996). Homology-facilitated illegitimate recombination between short regions has been observed in plastomes (Ogihara et al., 1988; Maréchal et al., 2009). Previous studies have shown that illegitimate recombination sites have GC-rich microhomologies of 3 bp to 10 bp (Prudhomme et al., 2002; De Vries et al., 2004). We present two hypotheses to explain how mitochondrial DNA can integrate into the plastome (**Figure 6**). In both hypotheses, identifying MIPT sequences adjacent to the transferred sequences in the *D. ibukiensis* mitogenome strongly indicated that homologous recombination plays a crucial role in facilitating the integration of its plastome. Both invasion and DSB are possible scenarios. The formation of a D-loop bubble is the predominant mechanism in plastid DNA and can facilitate homologous DNA pairing. The PLMT region in the *D. ibukiensis* plastome is associated with IR expansion, and DSB repair can occur via nonhomologous end-joining. Furthermore, identifying microhomologous sequences at the ends of the transferred sequences in the *D. ibukiensis* plastome suggested the possibility of illegitimate recombination. TE can be mobilized within a genome (Huang et al., 2012), and LTR retrotransposons can target tRNA genes. In the *D. ibukiensis* mitogenome, three TEs were detected around the transferred DNA, one within the transferred DNA, and two in either of the flanking regions (**Figure 6**), which were categorized as *gypsy* LTR retrotransposons. PLMT in the *D. ibukiensis* plastome is located upstream of the *trnH-GUG*. Thus, mitochondria-located PLMT may move from the mitogenome to the plastome. Two distinct mechanisms have been postulated to underlie these events: mitochondrial integration into the *D. carota* and *A. syriaca* plastomes were inferred by the likely mechanism of non-LTR retrotransposons and repair of a DSB by homologous recombination, respectively (Iorizzo et al., 2012; Straub et al., 2013). A physical connection between chloroplasts and mitochondria may facilitate the exchange of materials between these organelles. Stromules are dynamic tubular structures extending from the chloroplast surface of plant cells (Köhler et al., 1997; Hanson and Sattarzadeh, 2008). Stromules have been observed to interact with other organelles, including mitochondria, peroxisomes, and the endoplasmic reticulum (ER) (Hanson and Conklin, 2020), suggesting a potential role in inter-organelle communication and the exchange of molecules. The ER is a prevalent mediator in facilitating interactions among the other organelles (Mathur et al., 2022), suggesting that the delivered DNA may be able to be imported into the chloroplast through the ER. A previous study has shown that circular or linear foreign DNA can enter the chloroplast envelope under stressful environmental conditions (Cerutti and Jagendorf, 1995).

## Data availability statement

The original contributions presented in the study are publicly available. This data can be found here: National Center for Biotechnology Information (NCBI) GenBank, https://www.ncbi.nlm.nih.gov/genbank/, OR231235-OR231238, OR756216-OR756231, OR764728-OR764735, OR771027-OR771028, OR771084-OR771087.

## Author contributions

SJunP: Conceptualization, Data curation, Formal Analysis, Investigation, Methodology, Validation, Visualization, Writing - original draft. SJ-P: Conceptualization, Funding acquisition, Project administration, Writing - review & editing.
