# Supplementary material for: Intrageneric structural variation in organelle genomes from the genus Dystaenia (Apiaceae): genome rearrangement and mitochondrion-to-plastid DNA transfer
Source: Front Plant Sci. 2023 Dec 5;14:1283292. doi: 10.3389/fpls.2023.1283292 (PMC10728875; doi:10.3389/fpls.2023.1283292)
Supplement: Supplementary file 1 [file DataSheet_1.pdf]

Supplementary Material

Intrageneric structural variation in organelle genomes from the genus *Dystaenia* (Apiaceae): genome rearrangement and mitochondrion-to-plastid DNA transfer

Seongjun Park, SeonJoo Park*

*** Correspondence:** SeonJoo Park: sjpark01@ynu.ac.kr

Supplementary Data

Supplementary Figure 1. Graph showing the base per base depth of the sequencing coverage across the two *Dystaenia* plastid and mitochondrial genomes.

Supplementary Figure 2. Structural alignments of two *Dystaenia* plastid and mitochondrial genomes using Mauve.

Supplementary Figure 3. Assessment of the completeness of the transcriptomes using BUSCO.

Supplementary Figure 4. Amino acid sequence alignments of the nuclear-encoded mitochondrial targeted genes from two *Dystaenia*.

Supplementary Figure 5. Amino acid sequence alignments of the nuclear-encoded mitochondrial targeted *sdh4* genes from two *Dystaenia* with related species.

Supplementary Figure 6. Assay results using primers designed to amplify the inserted region.

Supplementary Figure 7. The inserted region in the plastid genome of *Dystaenia ibukiensis*.

Supplementary Table 1. The accession number for the nuclear-encoded genes for mitochondrion.

Supplementary Table 2. Predicted repeats of two *Dystaenia* plastomes.

Supplementary Table 3. Predicted repeats of two *Dystaenia* mitogenomes.

Supplementary Table 4. Transit peptide prediction of nuclear-encoded ORFs among the analyzed Apiaceae species

Supplementary Table 5. Plastid-derived DNA fragments in two *Dystaenia* mitogenomes.

Supplementary Table 6. Summary of putative transposable elements (TEs) in two *Dystaenia* mitogenomes.

Supplementary Figure 1. Graph showing the base per base depth of the sequencing cover age across the two *Dystaenia* plastid and mitochondrial genomes. Linear plastome and mitogenome maps are above each graph. Genes above and below the horizontal line correspond to the genes in Figures 2 and 3. The y-axis represents the sequencing depth on a logarithmic scale.

D. takeshimana plastome

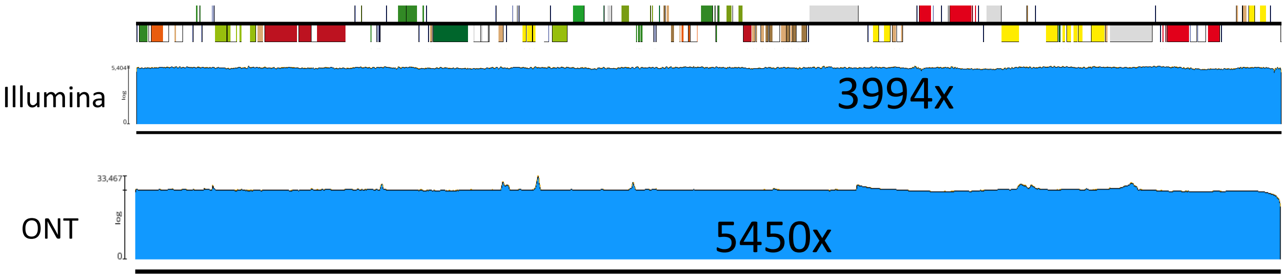

D. takeshimana mitogenome

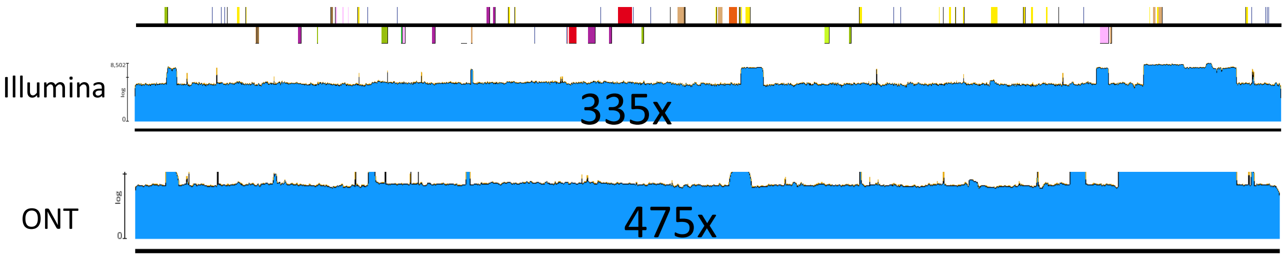

D. ibukiensis plastome

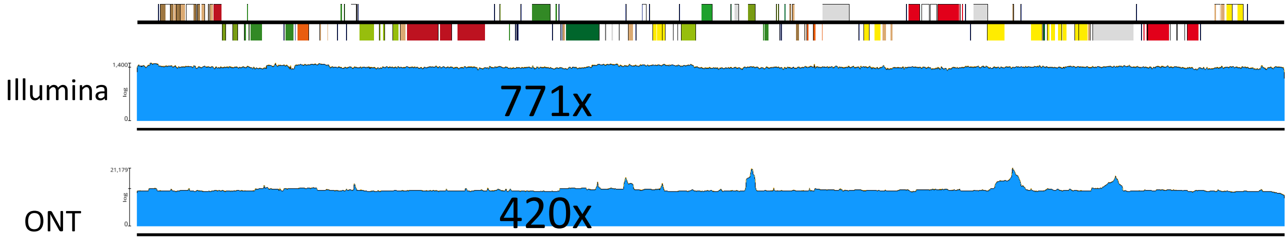

D. ibukiensis mitogenome

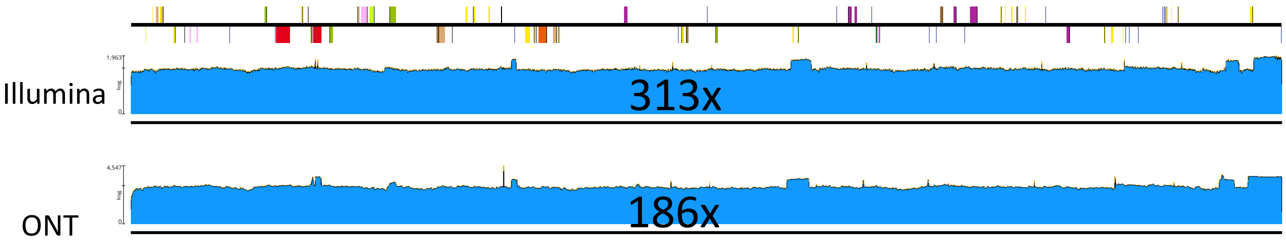

Supplementary Figure 2. Structural alignments of two *Dystaenia* plastid and mitochondrial genomes using Mauve. The colored block represents collinear sequence blocks shared by two plastomes (A) and two mitogenomes (B). The height of each bar reflects sequence similarity. The inverted repeat is shown for each plastome with pink boxes below each plastome block. (C) Mitochondrial gene clusters among two *Dystaenia*. The circles or X marks indicate the presence or absence of a gene cluster.

A

B

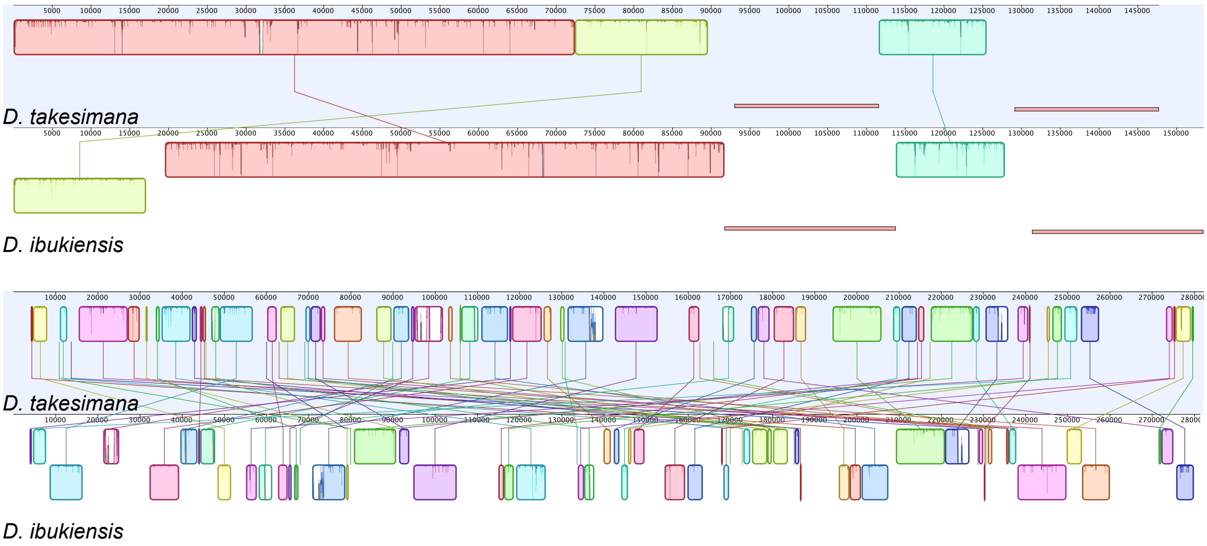

C

No.	gene clusters	*D. takeshimana*	*D. ibukiensis*
1	*atp8-cox3-sdh4*	×	×
2	*nad5 e3-nad1 e5*	×	×
3	*nad3-rps12*	◯	◯
4	*nad4L-atp4*	×	×
5	*rpl16-rps3-rps19-rpl2*	×	×
6	*cob-rps14-rpl5*	×	×
7	*rps10-cox1*	×	×
8	*rps13-nad1 e2-e3*	◯	◯
9	*rrn26- trnfM(CAU)*	◯	◯
10	*rrn18-rrn5*	◯	◯
11	*trnP(TGG)-sdh3*	×	×
12	*trnP(TGG)cp-trnW(CCA)cp*	×	×
13	*trnS(GCT)-trnF(GAA)-trnP(UGG)*	◯	◯
14	*trnY(GTA)-nad2 e5-e4-e3*	◯	◯
	Total	6	6

Supplementary Figure 3. Assessment of the completeness of the transcriptomes using BUSCO.

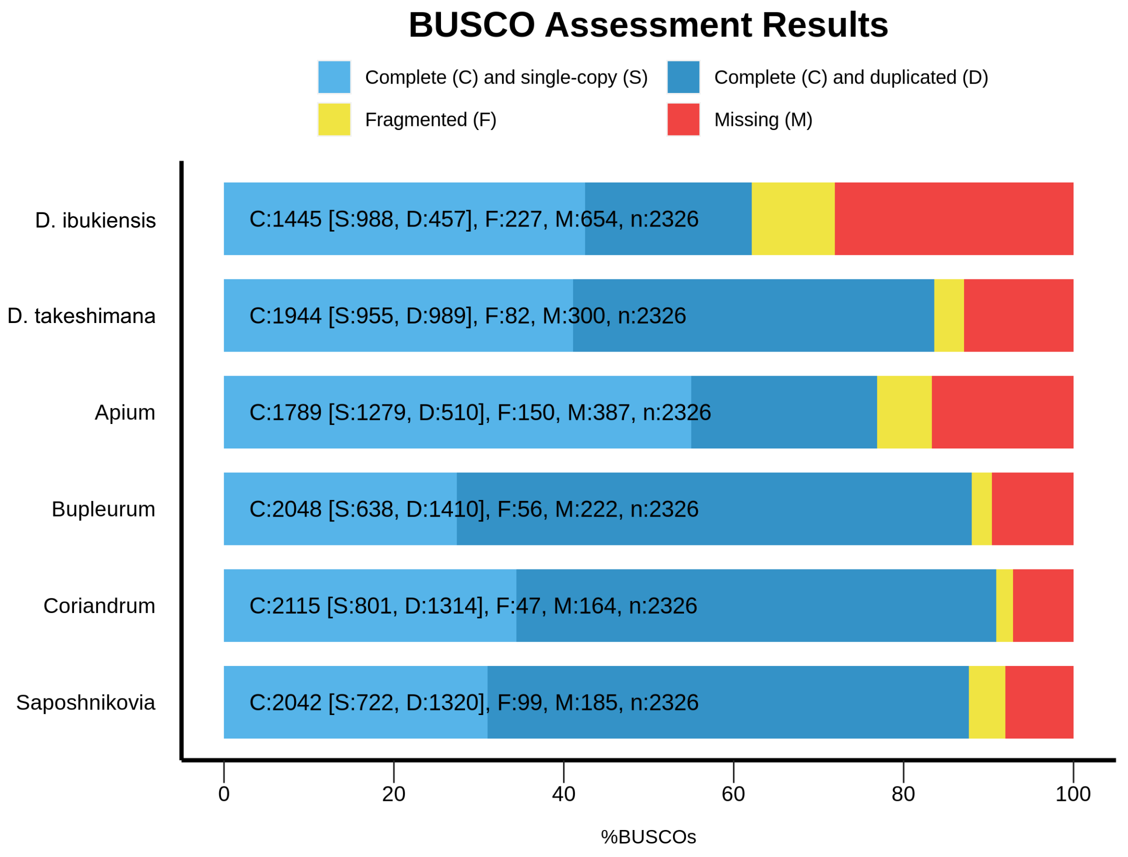

Supplementary Figure 4. Amino acid sequence alignments of the nuclear-encoded mitochondrial targeted genes from two *Dystaenia*. Boxes indicate mitochondrial targeting peptide (mTP: blue) and its conserved domains (red). A. 5′ *RPL2*, B. *RPS10*, C. *RPS14*, D. *RPS19*, E. *SDH3*. F. *SDH4*.

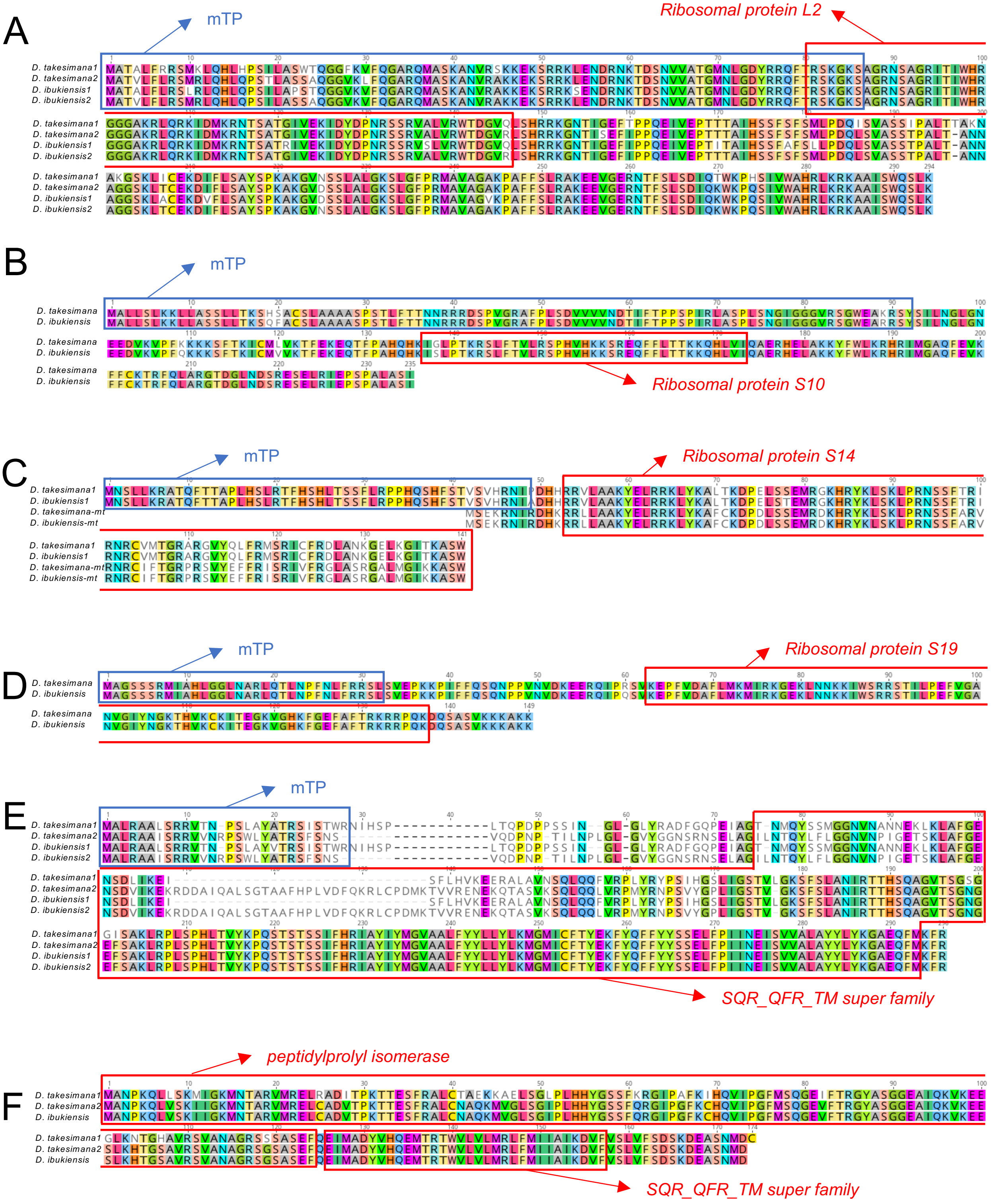

Supplementary Figure 5. Amino acid sequence alignments of the nuclear-encoded mitochondrial targeted *sdh4* genes from two *Dystaenia* with related species. Boxes indicate mitochondrial targeting peptide (mTP: blue) and its conserved domains (red).

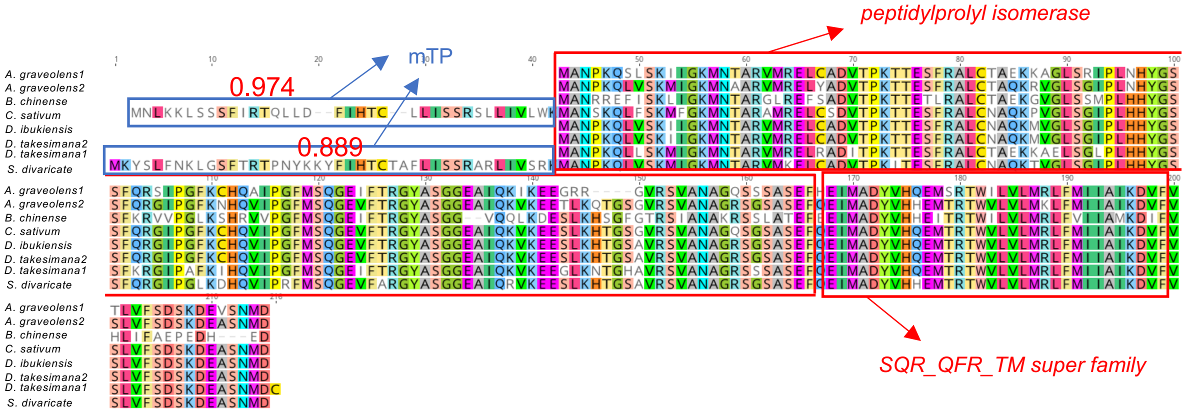

Supplementary Figure 6. Assay results using primers designed to amplify the inserted region. (A) A part map surrounding the inserted region in the *Dystaenia ibukiensis* plastome with the PCR amplicon sets. (B) Lane M contain the SolGent^{TM} 1 kb plus DNA ladder, lane 1 correspond to the PCR amplicons on A. plastid rbcL. For the plastid clpP gene, we designed a PCR screen that specifically targets the inserted region, which is expected to be 3,787 bp in size. If there is no inserted region, the product must be 1,377 bp in size.

A

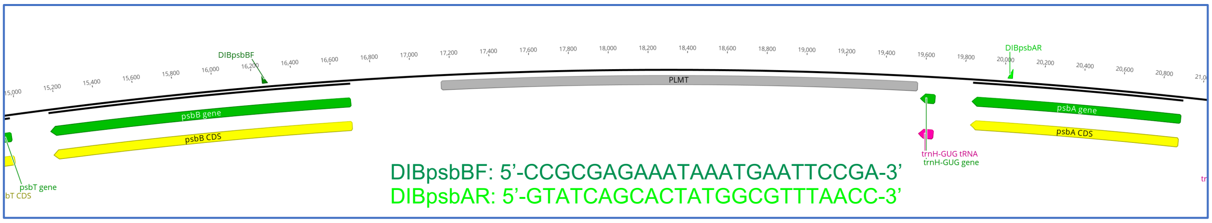

B

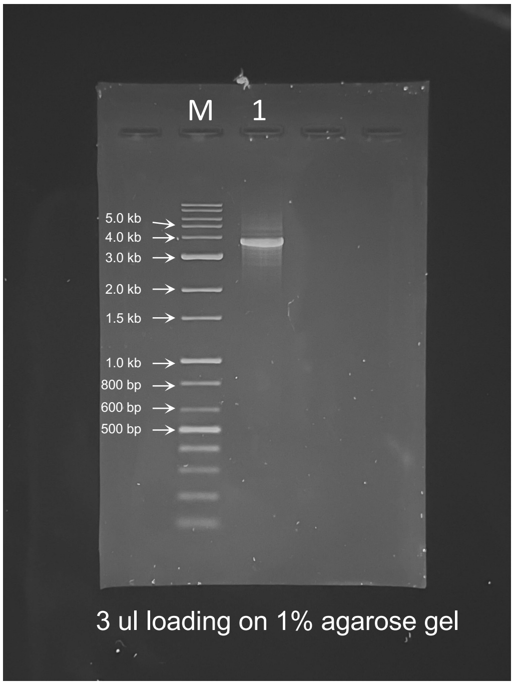

Supplementary Figure 7. The inserted region in the plastid genome of *Dystaenia ibukiensis*. Schematic diagram of the genomic region surrounding the inserted region in the D. ibukiensis plastome. Nucleotide sequences of the gray boxed region in the diagram . Colored boxes correspond to the genes in the diagram (top).

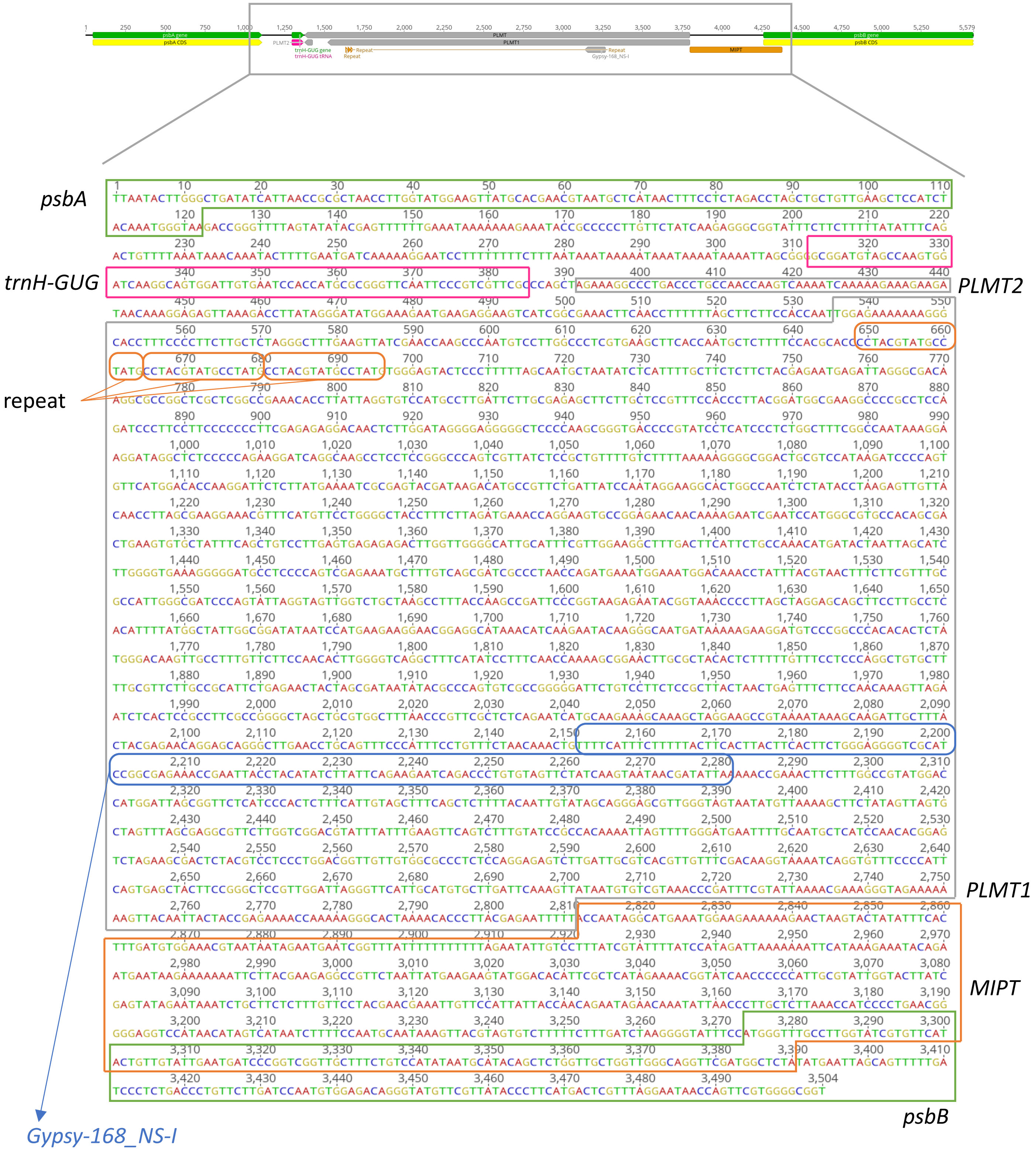

Supplementary Table 1. The accession number for the nuclear-encoded genes for mitochondrion.

Gene	Species	Accession number (genomic/transcriptomic)
5' RPL2	*D. takeshimana1*	OR756219 / OR764730
	D. takeshimana2	OR756220 / OR764731
	D. ibukiensis1	OR756217 / OR764728
	D. ibukiensis2	OR756218 / OR764729
RPS10	*D. takeshimana*	OR756222 / OR764733
	D. ibukiensis	OR756221 / OR764732
RPS14	*D. takeshimana1*	OR756224 / OR771028
	D. takeshimana2	OR756225 / -
	D. ibukiensis1	OR756223 / OR771027
	D. ibukiensis2	OR756216 / -
RPS19	*D. takeshimana*	OR756227 / OR764735
	D. ibukiensis	OR756226 / OR764734
SDH3	*D. takeshimana1*	OR756230 / OR771085
	D. takeshimana2	OR756231 / OR771087
	D. ibukiensis1	OR756228 / OR771084
	D. ibukiensis2	OR756229 / OR771086

Supplementary Table 2. Predicted repeats of two *Dystaenia* plastomes.

Species	Repeat	Length	Start	End	Direction
D. takeshimana	Repeat_1	41	91313	91353	plus
	Repeat_1	41	91331	91371	plus
	Repeat_2	38	29859	29896	plus
	Repeat_2	38	35316	35353	plus
	Repeat_3	34	31036	31069	plus
	Repeat_3	34	31593	31560	minus
	Repeat_4	33	12	44	plus
	Repeat_4	33	41	73	plus
	Repeat_5	32	30093	30124	plus
	Repeat_5	32	30124	30093	minus
	Repeat_6	30	8450	8479	plus
	Repeat_6	30	46445	46416	minus
D. ibukiensis	Repeat_1	51	17084	17134	plus
	Repeat_1	51	91729	91679	minus
	Repeat_2	41	93471	93511	plus
	Repeat_2	41	93489	93529	plus
	Repeat_2	41	151718	151678	minus
	Repeat_2	41	151736	151696	minus
	Repeat_3	34	95722	95755	plus
	Repeat_3	34	95761	95794	plus
	Repeat_3	34	149446	149413	minus
	Repeat_3	34	149485	149452	minus
	Repeat_4	34	50541	50574	plus
	Repeat_4	34	51098	51065	minus
	Repeat_5	32	49597	49628	plus
	Repeat_5	32	49628	49597	minus
	Repeat_6	32	19269	19300	plus
	Repeat_6	32	19285	19316	plus
	Repeat_7	30	27915	27944	plus
	Repeat_7	30	65420	65391	minus

Supplementary Table 3. Predicted repeats of two *Dystaenia* mitogenomes.

Species	Repeat	Length	Start	End	Direction
D. takeshimana	Repeat_1	5706	135481	141186	plus
	Repeat_1	5706	188491	194196	plus
	Repeat_2	4322	152836	157157	plus
	Repeat_2	4322	260369	264690	plus
	Repeat_3	3134	139498	142631	plus
	Repeat_3	3134	234174	237307	plus
	Repeat_4	2736	8034	10769	plus
	Repeat_4	2736	268253	265518	minus
	Repeat_5	2661	56811	59471	plus
	Repeat_5	2661	233202	235862	plus
	Repeat_6	1951	108965	110915	plus
	Repeat_6	1951	280261	282211	plus
	Repeat_7	1725	57783	59507	plus
	Repeat_7	1725	192508	194232	plus
	Repeat_8	1689	57783	59471	plus
	Repeat_8	1689	139498	141186	plus
	Repeat_8	1689	192508	194196	plus
	Repeat_8	1689	234174	235862	plus
	Repeat_9	1689	57783	59471	plus
	Repeat_9	1689	139498	141186	plus
	Repeat_9	1689	192508	194196	plus
	Repeat_9	1689	234174	235862	plus
	Repeat_10	1330	75141	76470	plus
	Repeat_10	1330	216037	217366	plus
	Repeat_11	1204	14184	15387	plus
	Repeat_11	1204	31990	33193	plus
	Repeat_12	1163	14395	15557	plus
	Repeat_12	1163	217029	215867	minus
	Repeat_13	1119	109797	110915	plus
	Repeat_13	1119	169305	168187	minus
	Repeat_13	1119	281093	282211	plus
	Repeat_14	1104	32201	33304	plus
	Repeat_14	1104	76133	75030	minus
	Repeat_15	1086	168135	169220	plus
	Repeat_15	1086	194248	193163	minus
	Repeat_15	1070	59507	58438	minus
	Repeat_16	1070	58438	59507	plus
	Repeat_16	1070	169220	168151	minus
	Repeat_16	1070	193163	194232	plus
	Repeat_17	1065	109882	110946	plus
	Repeat_17	1065	140153	141217	plus
	Repeat_17	1065	234829	235893	plus
	Repeat_18	1048	75209	76256	plus
	Repeat_18	1048	168951	167904	minus
	Repeat_18	1048	216105	217152	plus
	Repeat_19	1034	58438	59471	plus
	Repeat_19	1034	109882	110915	plus
	Repeat_19	1034	140153	141186	plus
	Repeat_19	1034	169220	168187	minus
	Repeat_19	1034	193163	194196	plus
	Repeat_19	1034	234829	235862	plus
	Repeat_19	1034	281178	282211	plus
	Repeat_20	1034	58438	59471	plus
	Repeat_20	1034	109882	110915	plus
	Repeat_20	1034	140153	141186	plus
	Repeat_20	1034	169220	168187	minus

Repeat_20	1034	193163	194196	plus
Repeat_20	1034	234829	235862	plus
Repeat_20	1034	281178	282211	plus
Repeat_21	993	14395	15387	plus
Repeat_21	993	32201	33193	plus
Repeat_21	993	76133	75141	minus
Repeat_21	993	217029	216037	minus
Repeat_22	993	14395	15387	plus
Repeat_22	993	32201	33193	plus
Repeat_22	993	76133	75141	minus
Repeat_22	993	217029	216037	minus
Repeat_23	925	14395	15319	plus
Repeat_23	925	32201	33125	plus
Repeat_23	925	76133	75209	minus
Repeat_23	925	168027	168951	plus
Repeat_23	925	217029	216105	minus
Repeat_24	817	14503	15319	plus
Repeat_24	817	32309	33125	plus
Repeat_24	817	76025	75209	minus
Repeat_24	817	168135	168951	plus
Repeat_24	817	194248	193432	minus
Repeat_24	817	216921	216105	minus
Repeat_25	801	14519	15319	plus
Repeat_25	801	32325	33125	plus
Repeat_25	801	59507	58707	minus
Repeat_25	801	76009	75209	minus
Repeat_25	801	168151	168951	plus
Repeat_25	801	194232	193432	minus
Repeat_25	801	216905	216105	minus
Repeat_26	765	14555	15319	plus
Repeat_26	765	32361	33125	plus
Repeat_26	765	59471	58707	minus
Repeat_26	765	75973	75209	minus
Repeat_26	765	110915	110151	minus
Repeat_26	765	141186	140422	minus
Repeat_26	765	168187	168951	plus
Repeat_26	765	194196	193432	minus
Repeat_26	765	216869	216105	minus
Repeat_26	765	235862	235098	minus
Repeat_26	765	282211	281447	minus
Repeat_27	384	1	384	plus
Repeat_27	384	168186	167803	minus
Repeat_28	335	34632	34966	plus
Repeat_28	335	66824	67158	plus
Repeat_29	283	168186	167904	minus
Repeat_29	283	1	283	plus
Repeat_29	283	75974	76256	plus
Repeat_29	283	216870	217152	plus
Repeat_30	191	82738	82928	plus
Repeat_30	191	279288	279098	minus
Repeat_31	160	1	160	plus
Repeat_31	160	75974	76133	plus
Repeat_31	160	216870	217029	plus
Repeat_31	160	14554	14395	minus
Repeat_31	160	32360	32201	minus
Repeat_31	160	168186	168027	minus
Repeat_32	147	73854	74000	plus
Repeat_32	147	105836	105690	minus

Repeat_33	101	121668	121768	plus
Repeat_33	101	142625	142725	plus
Repeat_34	84	115494	115577	plus
Repeat_34	84	141747	141830	plus
Repeat_34	84	236423	236506	plus
Repeat_35	78	45744	45821	plus
Repeat_35	78	214308	214385	plus
Repeat_36	75	229312	229238	minus
Repeat_36	75	176244	176318	plus
Repeat_37	71	143493	143563	plus
Repeat_37	71	157249	157179	minus
Repeat_38	71	62040	62110	plus
Repeat_38	71	106224	106294	plus
Repeat_39	64	92512	92575	plus
Repeat_39	64	113757	113820	plus
Repeat_40	60	29359	29418	plus
Repeat_40	60	30302	30361	plus
Repeat_41	57	108476	108532	plus
Repeat_41	57	143189	143133	minus
Repeat_42	56	79436	79491	plus
Repeat_42	56	233036	233091	plus
Repeat_43	54	92568	92515	minus
Repeat_43	54	113813	113760	minus
Repeat_43	54	79462	79515	plus
Repeat_44	54	14878	14931	plus
Repeat_44	54	32684	32737	plus
Repeat_44	54	59148	59095	minus
Repeat_44	54	75650	75597	minus
Repeat_44	54	110592	110539	minus
Repeat_44	54	138364	138417	plus
Repeat_44	54	140863	140810	minus
Repeat_44	54	168510	168563	plus
Repeat_44	54	191374	191427	plus
Repeat_44	54	193873	193820	minus
Repeat_44	54	216546	216493	minus
Repeat_44	54	235539	235486	minus
Repeat_44	54	281888	281835	minus
Repeat_45	53	138034	138086	plus
Repeat_45	53	191044	191096	plus
Repeat_45	53	245881	245829	minus
Repeat_46	52	1	52	plus
Repeat_46	52	14554	14503	minus
Repeat_46	52	32360	32309	minus
Repeat_46	52	75974	76025	plus
Repeat_46	52	168186	168135	minus
Repeat_46	52	194197	194248	plus
Repeat_46	52	216870	216921	plus
Repeat_47	50	59119	59070	minus
Repeat_47	50	75621	75572	minus
Repeat_47	50	110563	110514	minus
Repeat_47	50	140834	140785	minus
Repeat_47	50	193844	193795	minus
Repeat_47	50	216517	216468	minus
Repeat_47	50	235510	235461	minus
Repeat_47	50	281859	281810	minus
Repeat_47	50	14907	14956	plus
Repeat_47	50	32713	32762	plus
Repeat_47	50	138313	138362	plus

Repeat_47	50	168539	168588	plus
Repeat_47	50	191323	191372	plus
Repeat_48	49	84041	84089	plus
Repeat_48	49	141737	141689	minus
Repeat_48	49	236413	236365	minus
Repeat_49	49	211873	211825	minus
Repeat_49	49	80068	80116	plus
Repeat_50	48	132625	132672	plus
Repeat_50	48	274465	274418	minus
Repeat_51	46	14541	14586	plus
Repeat_51	46	32347	32392	plus
Repeat_51	46	59485	59440	minus
Repeat_51	46	75987	75942	minus
Repeat_51	46	105831	105876	plus
Repeat_51	46	168173	168218	plus
Repeat_51	46	194210	194165	minus
Repeat_51	46	216883	216838	minus
Repeat_52	45	34631	34675	plus
Repeat_52	45	58429	58473	plus
Repeat_52	45	140144	140188	plus
Repeat_52	45	193154	193198	plus
Repeat_52	45	234820	234864	plus
Repeat_53	44	34632	34675	plus
Repeat_53	44	58430	58473	plus
Repeat_53	44	66824	66867	plus
Repeat_53	44	140145	140188	plus
Repeat_53	44	193155	193198	plus
Repeat_53	44	234821	234864	plus
Repeat_54	44	6900	6943	plus
Repeat_54	44	273322	273365	plus
Repeat_55	43	125193	125235	plus
Repeat_55	43	157145	157187	plus
Repeat_56	41	269608	269648	plus
Repeat_56	41	269626	269666	plus
Repeat_57	41	44367	44407	plus
Repeat_57	41	108513	108553	plus
Repeat_58	40	99721	99760	plus
Repeat_58	40	124473	124512	plus
Repeat_59	39	12075	12113	plus
Repeat_59	39	30590	30628	plus
Repeat_60	38	6945	6982	plus
Repeat_60	38	273367	273404	plus
Repeat_61	37	123861	123897	plus
Repeat_61	37	123870	123906	plus
Repeat_62	37	122305	122341	plus
Repeat_62	37	202642	202678	plus
Repeat_63	36	107210	107245	plus
Repeat_63	36	148308	148343	plus
Repeat_64	36	169220	169185	minus
Repeat_64	36	34640	34675	plus
Repeat_64	36	58438	58473	plus
Repeat_64	36	66832	66867	plus
Repeat_64	36	109882	109917	plus
Repeat_64	36	140153	140188	plus
Repeat_64	36	193163	193198	plus
Repeat_64	36	234829	234864	plus
Repeat_64	36	281178	281213	plus
Repeat_65	36	1	36	plus

	Repeat_65	36	59472	59507	plus
	Repeat_65	36	75974	76009	plus
	Repeat_65	36	194197	194232	plus
	Repeat_65	36	216870	216905	plus
	Repeat_65	36	14554	14519	minus
	Repeat_65	36	32360	32325	minus
	Repeat_65	36	168186	168151	minus
	Repeat_66	35	108185	108219	plus
	Repeat_66	35	259677	259711	plus
	Repeat_67	34	98254	98287	plus
	Repeat_67	34	274894	274861	minus
	Repeat_68	33	107581	107613	plus
	Repeat_68	33	259069	259101	plus
	Repeat_69	33	95460	95492	plus
	Repeat_69	33	95487	95519	plus
	Repeat_70	33	215681	215649	minus
	Repeat_70	33	73041	73073	plus
	Repeat_71	32	14555	14586	plus
	Repeat_71	32	32361	32392	plus
	Repeat_71	32	59471	59440	minus
	Repeat_71	32	75973	75942	minus
	Repeat_71	32	105845	105876	plus
	Repeat_71	32	110915	110884	minus
	Repeat_71	32	141186	141155	minus
	Repeat_71	32	168187	168218	plus
	Repeat_71	32	194196	194165	minus
	Repeat_71	32	216869	216838	minus
	Repeat_71	32	235862	235831	minus
	Repeat_71	32	282211	282180	minus
	Repeat_72	32	10761	10792	plus
	Repeat_72	32	265463	265494	plus
	Repeat_73	31	128718	128748	plus
	Repeat_73	31	128760	128790	plus
	Repeat_74	31	274859	274829	minus
	Repeat_74	31	98289	98319	plus
	Repeat_75	31	96385	96415	plus
	Repeat_75	31	96421	96451	plus
	Repeat_76	31	92423	92453	plus
	Repeat_76	31	274312	274282	minus
	Repeat_77	31	6945	6975	plus
	Repeat_77	31	49020	49050	plus
	Repeat_77	31	273367	273397	plus
	Repeat_78	30	278645	278674	plus
	Repeat_78	30	278716	278745	plus
	Repeat_79	30	79491	79462	minus
	Repeat_79	30	233091	233062	minus
	Repeat_79	30	92539	92568	plus
	Repeat_79	30	113784	113813	plus
D. ibukiensis	Repeat_1	6502	68195	74696	plus
	Repeat_1	6502	188807	195308	plus
	Repeat_2	5280	106339	111618	plus
	Repeat_2	5280	263382	268661	plus
	Repeat_3	4099	24804	28902	plus
	Repeat_3	4099	154504	150406	minus
	Repeat_4	3569	1	3569	plus
	Repeat_4	3569	63007	59439	minus
	Repeat_5	3556	105623	109178	plus
	Repeat_5	3556	136381	139936	plus

Repeat_6	2840	106339	109178	plus
Repeat_6	2840	137097	139936	plus
Repeat_6	2840	263382	266221	plus
Repeat_7	2836	105773	108608	plus
Repeat_7	2836	136531	139366	plus
Repeat_7	2836	235295	232460	minus
Repeat_8	2553	29817	32369	plus
Repeat_8	2553	105928	108480	plus
Repeat_8	2553	136686	139238	plus
Repeat_8	2553	235140	232588	minus
Repeat_9	2270	108608	106339	minus
Repeat_9	2270	139366	137097	minus
Repeat_9	2270	232460	234729	plus
Repeat_9	2270	265651	263382	minus
Repeat_10	2270	148306	150575	plus
Repeat_10	2270	223900	226169	plus
Repeat_11	2244	60868	63111	plus
Repeat_11	2244	261242	263485	plus
Repeat_12	2142	30228	32369	plus
Repeat_12	2142	106339	108480	plus
Repeat_12	2142	137097	139238	plus
Repeat_12	2142	234729	232588	minus
Repeat_12	2142	263382	265523	plus
Repeat_13	2140	1	2140	plus
Repeat_13	2140	63007	60868	minus
Repeat_13	2140	263381	261242	minus
Repeat_14	704	94928	95631	plus
Repeat_14	704	183948	184651	plus
Repeat_15	170	28733	28902	plus
Repeat_15	170	150575	150406	minus
Repeat_15	170	226169	226000	minus
Repeat_16	104	30228	30331	plus
Repeat_16	104	63008	63111	plus
Repeat_16	104	106339	106442	plus
Repeat_16	104	137097	137200	plus
Repeat_16	104	234729	234626	minus
Repeat_16	104	263382	263485	plus
Repeat_17	101	35928	36028	plus
Repeat_17	101	105015	105115	plus
Repeat_18	95	51733	51827	plus
Repeat_18	95	124291	124385	plus
Repeat_19	83	16384	16466	plus
Repeat_19	83	135332	135414	plus
Repeat_20	70	8007	8076	plus
Repeat_20	70	30623	30692	plus
Repeat_20	70	106734	106803	plus
Repeat_20	70	137492	137561	plus
Repeat_20	70	263777	263846	plus
Repeat_20	70	234334	234265	minus
Repeat_21	66	51666	51731	plus
Repeat_21	66	124224	124289	plus
Repeat_22	66	30140	30205	plus
Repeat_22	66	30211	30276	plus
Repeat_22	66	106251	106316	plus
Repeat_22	66	106322	106387	plus
Repeat_22	66	137009	137074	plus
Repeat_22	66	137080	137145	plus
Repeat_22	66	234746	234681	minus

Repeat_22	66	234817	234752	minus
Repeat_23	64	42371	42434	plus
Repeat_23	64	204727	204664	minus
Repeat_24	58	154303	154246	minus
Repeat_24	58	25005	25062	plus
Repeat_24	58	70403	70460	plus
Repeat_24	58	191015	191072	plus
Repeat_25	57	46509	46565	plus
Repeat_25	57	104551	104607	plus
Repeat_26	56	256680	256625	minus
Repeat_26	56	223434	223489	plus
Repeat_27	55	23197	23251	plus
Repeat_27	55	172418	172472	plus
Repeat_28	54	42374	42427	plus
Repeat_28	54	256601	256654	plus
Repeat_28	54	204724	204671	minus
Repeat_29	50	33158	33207	plus
Repeat_29	50	185030	185079	plus
Repeat_30	49	256048	256000	minus
Repeat_30	49	162241	162289	plus
Repeat_31	49	30157	30205	plus
Repeat_31	49	30228	30276	plus
Repeat_31	49	63008	63056	plus
Repeat_31	49	106268	106316	plus
Repeat_31	49	106339	106387	plus
Repeat_31	49	137026	137074	plus
Repeat_31	49	137097	137145	plus
Repeat_31	49	234729	234681	minus
Repeat_31	49	234800	234752	minus
Repeat_31	49	263382	263430	plus
Repeat_32	48	272035	272082	plus
Repeat_32	48	272051	272098	plus
Repeat_33	48	77508	77555	plus
Repeat_33	48	134311	134264	minus
Repeat_34	38	188001	188038	plus
Repeat_34	38	203272	203309	plus
Repeat_35	38	49428	49465	plus
Repeat_35	38	135325	135362	plus
Repeat_36	37	146524	146560	plus
Repeat_36	37	146554	146590	plus
Repeat_37	37	80818	80854	plus
Repeat_37	37	169910	169874	minus
Repeat_38	37	35355	35391	plus
Repeat_38	37	217688	217652	minus
Repeat_39	36	99429	99394	minus
Repeat_39	36	45243	45278	plus
Repeat_40	36	22971	23006	plus
Repeat_40	36	23051	23086	plus
Repeat_41	35	249779	249745	minus
Repeat_41	35	168312	168346	plus
Repeat_42	34	68861	68894	plus
Repeat_42	34	68903	68936	plus
Repeat_42	34	189473	189506	plus
Repeat_42	34	189515	189548	plus
Repeat_43	34	24165	24198	plus
Repeat_43	34	133835	133868	plus
Repeat_44	33	173007	173039	plus
Repeat_44	33	270875	270907	plus

Repeat_45	33	115344	115376	plus
Repeat_45	33	229061	229093	plus
Repeat_46	33	21540	21572	plus
Repeat_46	33	21567	21599	plus
Repeat_47	32	272035	272066	plus
Repeat_47	32	272051	272082	plus
Repeat_47	32	272067	272098	plus
Repeat_48	32	73342	73373	plus
Repeat_48	32	193954	193985	plus
Repeat_48	32	270467	270498	plus
Repeat_49	31	42282	42312	plus
Repeat_49	31	134417	134447	plus
Repeat_50	31	24200	24230	plus
Repeat_50	31	133870	133900	plus
Repeat_51	31	16384	16414	plus
Repeat_51	31	49435	49465	plus
Repeat_51	31	135332	135362	plus
Repeat_52	30	252735	252764	plus
Repeat_52	30	252806	252835	plus
Repeat_53	30	204700	204671	minus
Repeat_53	30	223489	223460	minus
Repeat_53	30	42398	42427	plus
Repeat_53	30	256625	256654	plus
Repeat_54	30	146642	146613	minus
Repeat_54	30	232639	232610	minus
Repeat_54	30	32318	32347	plus
Repeat_54	30	108429	108458	plus
Repeat_54	30	139187	139216	plus
Repeat_54	30	265472	265501	plus
Repeat_55	30	22889	22918	plus
Repeat_55	30	68314	68343	plus
Repeat_55	30	188926	188955	plus

Supplementary Table 4. Transit peptide prediction of nuclear-encoded ORFs among the analyzed Apiaceae species.

Gene	Speceis	TargetP-2.0					Predotar v1.04		LOCALIZER 1.0.4			
		length (aa)	cTP	mTP	Probability	Tplen	Plastid	Mitochondrial	mTP		cTP	
5' RPL2	*Saposhnikovia1*	294	0.0008	**0.9764**	0.6189	37	0	**0.86**	**0.999**	21	-	-
	Saposhnikovia2	293	0.0018	**0.9754**	0.5275	37	0.03	**0.9**	**0.999**	21	-	-
	Coriandrum	294	0.0040	**0.9451**	0.5382	37	0.11	**0.79**	**0.997**	21	-	-
	Apium	293	0.0060	**0.9589**	0.5751	37	0.01	**0.43**	**0.997**	21	-	-
	Daucus	293	0.0020	**0.9286**	0.5227	37	0.02	**0.88**	**0.997**	21	-	-
	Bupleurum	294	0.0007	**0.8094**	0.5676	37	0.07	**0.69**	**0.992**	25	-	-
RPS10	*Saposhnikovia*	211	0.3234	**0.5929**	0.1852	77	0.02	**0.83**	**0.953**	22	-	-
	Daucus1	238	**0.5916**	0.3171	0.1586	45	0.52	0.46	**0.883**	48	**0.809**	33
	Daucus2	231	0.3446	**0.5857**	0.2879	89	0.49	0.47	**0.818**	28	**0.958**	22
RPS14	*Saposhnikovia*	144	0.1463	**0.7536**	0.5628	44	0.06	0.77	**0.981**	35	**0.927**	35
	Coriandrum	133	0.0314	**0.5845**	0.3863	31	0.08	**0.57**	**0.974**	22	**0.96**	41
	Apium	144	0.1872	**0.4658**	0.489	42	0.11	**0.25**	0.966	33	**0.969**	35
	Daucus	158	0.0645	**0.8956**	0.3984	56	0.02	**0.76**	**0.997**	23	-	-
RPS19	*Saposhnikovia*	150	0.0717	**0.7736**	0.3402	32	0.03	**0.45**	-	-	**0.997**	32
	Coriandrum	158	0.1325	**0.6897**	0.2337	32	0.02	**0.31**	0.958	21	**0.998**	41
	Apium	152	0.0122	**0.7863**	0.2265	28	0	**0.49**	**0.98**	30	-	-
	Daucus	153	0.1656	**0.4259**	0.3928	44	0.05	**0.34**	**0.957**	30	-	-
	Bupleurum	151	0.0102	**0.8964**	0.2157	37	0.06	**0.52**	**0.984**	26	0.825	26
SDH3	*Saposhnikovia1*	250	0.0007	**0.9959**	0.1862	22	0	**0.83**	**0.997**	25	-	-
	Saposhnikovia2	274	0.0001	**0.9982**	0.4049	24	0	**0.84**	**0.998**	23	-	-
	Coriandrum1	259	0.0002	**0.9949**	0.1952	23	0	**0.81**	**0.995**	27	-	-
	Coriandrum2	277	0.0002	**0.9948**	0.2896	23	0	**0.79**	**0.998**	24	-	-
	Apium1	228	0.0014	**0.9929**	0.3551	28	0	**0.74**	**0.996**	28	-	-
	Apium2	276	0.0000	**0.9972**	0.2765	24	0	**0.9**	**1.000**	21	-	-
	Daucus	255	0.0003	**0.9974**	0.3599	34	0	**0.87**	**0.998**	28	-	-
	Bupleurum	231	0.0004	**0.9978**	0.4404	26	0	**0.86**	**0.998**	26	-	-

Supplementary Table 5. Plastid-derived DNA fragments in two *Dystaenia* mitogenomes.

Species	Identity	Aligned length	number of mismatches	number of gap openings	Query start	Query end	Hit start	Hit end	E value	Bit-score	Annotation
D. takeshimana	100	578	0	0	39444	40021	85347	85924	0	1068	psaB"
	100	79	0	0	104	182	57121	57043	2.03E-34	147	trnH-GUG
	100	79	0	0	104	182	233512	233434	2.03E-34	147	trnH-GUG
	99.994	17611	1	0	90137	107747	270842	253232	0	32516	23S, trnA-UGC, trnI-GAU, 16S, trnV-GAC, rps12, rps7, ndhB, trnL-CAA, ycf2*
	99.977	4325	1	0	96289	100613	157157	152833	0	7982	ndhB* rps7 rps12
	99.96	2476	1	0	22884	25359	241978	244453	0	4567	rpoC1", rpoB"
	99.927	2742	2	0	92720	95461	8028	10769	0	5053	ycf2*, trnL-CAA, ndhB*
	98.78	82	1	0	109241	109322	21043	20962	2.03E-34	147	trnN-GUU
	98.148	54	1	0	41694	41747	13302	13355	7.47E-19	95.3	psaA*
	98.14	215	4	0	25502	25716	185739	185953	2.38E-103	375	rpoB*
	94.231	52	3	0	91324	91375	269691	269640	2.09E-14	80.5	ycf2*
	93.939	66	4	0	66782	66847	98254	98319	1.61E-20	100	trnW-CCA
	91.026	78	4	1	86665	86739	72872	72949	4.47E-21	102	trnI-CAU
	90	160	15	1	30986	31144	64528	64369	3.31E-52	206	trnD-GUC
	89.706	68	7	0	103019	103086	78172	78105	1.25E-16	87.9	trnI-GAU*
	86.885	61	8	0	101005	101065	108523	108463	4.53E-11	69.4	16S*
	84.96	379	43	6	66504	66876	275170	274800	3.08E-102	372	petG*, trnW-CCA
	83.158	95	16	0	107443	107537	142631	142725	1.25E-16	87.9	23S*
	80.412	97	19	0	105353	105449	119548	119644	9.74E-13	75	23S*
D. ibukiensis	93.939	66	4	0	85993	86058	24165	24230	1.63E-20	100	trnW-CCA

99.275	1103	5	1	1789	2888	94942	93840	0	1989	trnI-CAUp
83.158	95	16	0	109644	109738	105109	105015	1.27E-16	87.9	23S*
80.583	103	18	2	50217	50319	119106	119006	7.64E-14	78.7	psbM-trnD-GUC spacer*
92.453	106	8	0	85982	86087	133824	133929	4.44E-36	152	trnW-CCA
96.774	62	2	0	19525	19586	143770	143709	1.26E-21	104	trnH-GUG
100	4326	0	0	20075	24400	163498	167823	0	7989	psbA* trnK, matK
89.375	160	16	1	50491	50649	181395	181236	1.56E-50	200	trnD-GUC
93.953	215	13	0	44996	45210	198020	197806	2.47E-88	326	rpoB*
100	83	0	0	19573	19655	223908	223826	1.23E-36	154	trnH
97.561	82	2	0	111442	111523	244268	244349	9.60E-33	141	trnN-GUU
91.176	68	6	0	105219	105286	257951	258018	2.73E-18	93.5	trnI-GAU*
99.443	509	0	1	16575	19430	269341	269919	0	5201	clpP*-psbB*
96.7	5791	69	23	105682	110233	275728	281431	0	8107	trnI-GAU*, trnA-UGC, 23S, 4.5S, 5S, trnR-ACG, trnN-GUU*

Supplementary Table 6. Summary of putative transposable elements (TEs) in two *Dystaenia* mitogenomes.

Summary Table	*D. takeshimana*		*D. ibukiensis*	
Repeat Class	Fragments	Length	Fragments	Length
Integrated Virus	4	244	2	110
Caulimoviridae	4	244	2	110
Multicopy gene	7	443	8	485
tRNA	7	443	8	485
Simple Repeat	2	135	2	135
Satellite	2	135	2	135
SAT	1	66	1	66
Transposable Element	178	16,272	173	15,522
DNA transposon	82	6,823	79	6,305
EnSpm/CACTA	16	1,294	17	994
Harbinger	6	372	3	197
Helitron	17	1,568	20	1,933
Mariner/Tc1	3	227	2	152
MuDR	26	2,207	25	2,179
hAT	13	1,050	12	850
LTR Retrotransposon	81	8,095	82	8,069
Copia	30	2,414	29	2,292
Gypsy	48	4,918	47	4,878
Non-LTR Retrotransposon	15	1,354	12	1,148
L1	14	1,289	11	1,083
RTEX	1	65	1	65
Total	191	17,094	185	16,252
